# Spatial transcriptomics reveals immune-stromal crosstalk within the synovium of patients with juvenile idiopathic arthritis

**DOI:** 10.1172/jci.insight.198074

**Published:** 2025-11-21

**Authors:** Jun Inamo, Roselyn Fierkens, Michael R. Clay, Anna Helena Jonsson, Clara Lin, Kari Hayes, Nathan Rogers, Heather Leach, Kentaro Yomogida

**Affiliations:** 1 Department of Biomedical Informatics, Center for Health Artificial Intelligence, and; 2 Division of Rheumatology, University of Colorado School of Medicine, Aurora, Colorado, USA.; 3 Division of Rheumatology, Department of Internal Medicine, Keio University School of Medicine, Shinjuku-ku, Tokyo, Japan.; 4 Division of Rheumatology, Department of Pediatrics,; 5 Department of Pathology,; 6 Department of Radiology, and; 7 Department of Pediatrics, University of Colorado School of Medicine, Aurora, Colorado, USA.; 8 Colorado Child Health Research Institute, Children’s Hospital Colorado, Aurora, Colorado, USA.

**Keywords:** Autoimmunity, Immunology, Autoimmune diseases

## Abstract

Juvenile idiopathic arthritis (JIA) is the most prevalent chronic inflammatory arthritis of childhood, yet the spatial organization in the synovium remains poorly understood. Here, we perform subcellular-resolution spatial transcriptomic profiling of synovial tissue from patients with active JIA. We identify diverse immune and stromal cell populations and reconstruct spatially defined cellular niches. Applying a newly developed spatial colocalization analysis pipeline, we uncover microanatomical structures, including endothelial-fibroblast interactions mediated by NOTCH signaling, and a CXCL9/CXCR3 signaling axis between inflammatory macrophages and CD8^+^ T cells, alongside the characterization of other resident macrophage subsets. We also detect and characterize tertiary lymphoid structures marked by CXCL13/CXCR5 and CCL19-mediated signaling from Tph cells and immunoregulatory DCs, analogous to those observed in other autoimmune diseases. Finally, comparative analysis with rheumatoid arthritis reveals JIA-enriched cell states, including *NOTCH3^+^* and *CXCL12^+^* sublining fibroblasts, suggesting potentially differential inflammatory programs in pediatric versus adult arthritis. These findings provide a spatially resolved molecular framework of JIA synovitis and introduce a generalizable computational pipeline for spatial colocalization analysis in tissue inflammation.

## Abstract 2

Spatial transcriptomics reveals immune–stromal niches and disease-specific interactions in juvenile idiopathic arthritis synovial tissue.

## Graphical abstract

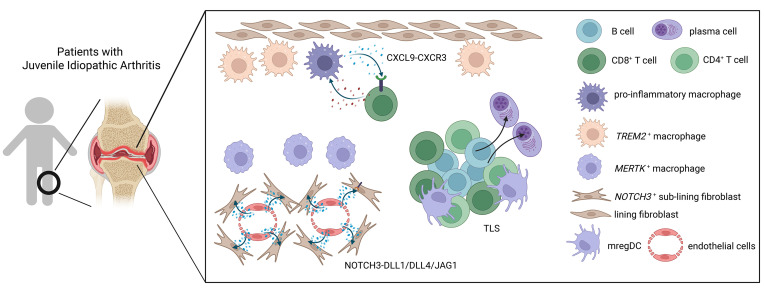

## Introduction

Juvenile idiopathic arthritis (JIA) is the most common chronic arthritis of childhood, affecting 1.6–23 cases per 100,000 children (1, 2). It comprises a heterogeneous group of conditions classified into 8 subtypes (3), of which oligoarticular and rheumatoid factor–negative (RF-negative) polyarticular JIA (hereafter referred to as oligo/poly JIA) account for 60%–80% of cases in North America (4). Recent advances in therapy have transformed the overall prognosis of JIA; however, a substantial proportion of children with oligo/poly JIA continue to experience chronic, relapsing disease courses (5, 6). The biological processes that determine the prognosis remain largely unknown.

Synovial tissue, a connective tissue lining the joints, is the primary site of the inflammation in JIA. However, previous studies on JIA have relied on synovial fluid due to practical difficulties of obtaining tissue. A recent study provided a cellular atlas of treatment-naive JIA synovium, combining CITE-Seq with in situ spatial transcriptomics platform that profiled 377 genes (7). This study highlighted the age-dependent differences in synovial cell populations and identified macrophage subsets associated with disease progression.

To deepen these insights, we sought to generate a comprehensive spatial and transcriptional profile of the JIA synovium. In this study, we applied a high-plex in situ transcriptomic platform (10x Genomics Xenium Prime 5K) to JIA synovial tissue, enabling the transcriptomes of thousands of cells to be measured within their native spatial organization at near single-cell (sub-10 μm) resolution in formalin-fixed, paraffin-embedded (FFPE) tissues. We developed an analytic pipeline tailored for high-resolution spatial transcriptome data, performing spatial neighborhood enrichment analyses at both cell type and single-spot levels. Using this approach, we rediscovered the spatial interaction between fibroblasts and endothelial cells — previously identified in rheumatoid arthritis (RA) synovium (8) — in JIA tissues. Furthermore, we characterized macrophage and T cell cross-talk and defined tertiary lymphoid structures (TLS) within the JIA synovial microenvironment, providing insights into immune-stromal interactions in JIA.

## Results

### High-resolution spatial profiling uncovers cellular architecture in JIA synovium.

To explore the cellular diversity and spatial organization within the inflamed synovium of patients with JIA, we employed advanced spatial transcriptomic profiling on synovial biopsy samples from 9 patients with oligo/poly JIA, defined by International League of Associations for Rheumatology (ILAR) classification (3) (Supplemental Table 1; supplemental material available online with this article; https://doi.org/10.1172/jci.insight.198074DS1). All oligoarticular patients with JIA were classified as a persistent subtype at the time of biopsy. We collected synovial biopsies from treatment-naive patients (*n* = 3) and from patients who had received various treatments, including intraarticular steroid injections, methotrexate, leflunomide, and anti-TNF inhibitors (*n* = 6). All patients were negative for RF. Six patients have normal C-reactive protein (CRP) levels, a finding consistent with previous reports indicating that 50%–70% of patients with oligo/poly JIA exhibit normal CRP (9, 10). At the time of biopsy, all of our JIA samples exhibited active synovitis, as diagnosed by board-certified pediatric rheumatologists and supported by elevated Krenn inflammatory infiltrate scores (Supplemental Table 1).

**Figure 1:**
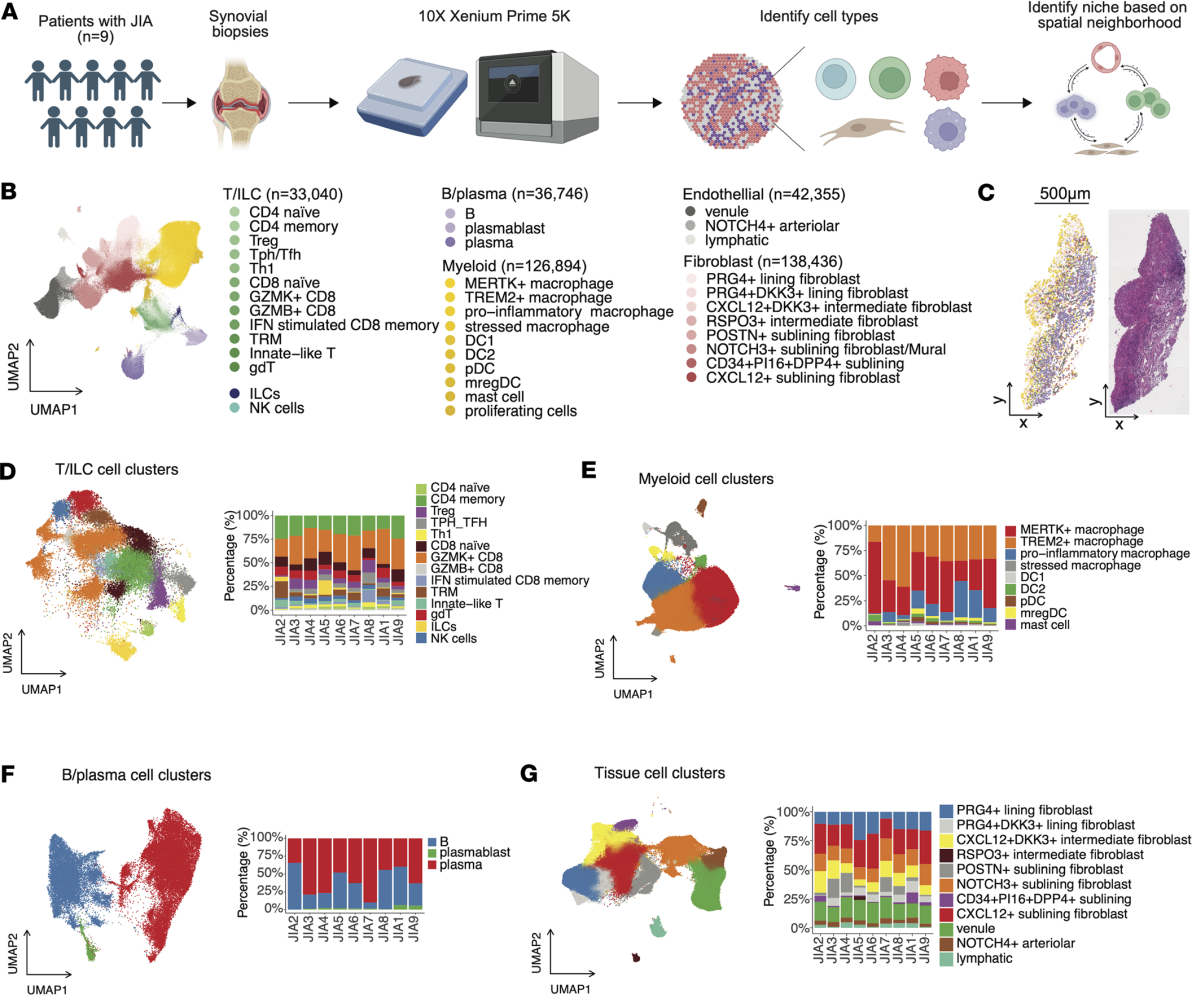
Spatial transcriptomic profiling of synovial tissue in patients with JIA. (**A**) Synovial biopsy samples were collected from 9 patients diagnosed with JIA. Subcellular resolution spatial transcriptomic data were acquired from FFPE biopsy samples using the 10X Xenium Prime 5K platform. Cell types within synovial tissues were identified, and spatial neighborhoods were characterized based on spatial proximity analysis. (**B**) Identified major immune and tissue-associated cellular compartments in synovium, including T cells, B cells, myeloid cells, and stromal tissue cells (endothelial and fibroblast subsets). Within each broad cell type, fine-scale cell subpopulations were annotated. (**C**) Representative example of the FFPE histological slide of synovial biopsy sample and spatial mapping of identified cell clusters. Colors are corresponding with panel **B**. Scale bar: 500 μm. (**D**–**G**) Identified fine-scaled cell clusters on UMAP (left) and composition across samples (right) for T cells (**D**), myeloid cells (**E**), B/plasma cells (**F**), and stromal tissue cells (**G**).

Utilizing the 10X Xenium Prime 5K platform, we generated high-resolution spatial transcriptomic data capable of identifying discrete cell populations and characterizing their spatial interactions and niche formations within the tissue microenvironment (Figure 1A and Supplemental Figures 1 and 2). After preprocessing and batch correction steps (Supplemental Figure 1, A–C), we performed unsupervised clustering of the spatial transcriptomic data and, based on manual annotation using canonical markers, delineated 4 major cellular compartments: T cell–innate lymphoid cells (ILCs), B/plasma cells, myeloid cells, and tissue-associated stromal cells, which included diverse subsets of fibroblasts and endothelial cells (Figure 1, B–G). Marker genes for each subcluster are listed in Supplemental Table 2, and spatial visualization of annotated cell clusters on histological sections are provided in Supplemental Data 1–9 in an interactive format.

T cell–ILC subpopulations included fine-scaled T cell subsets as well as ILCs (Supplemental Figure 1D). The CD8^+^ T cell population encompasses *CCR7*^+^*TCF7*^+^ naive CD8^+^ T cells, along with granzyme B^+^ (*GZMB*^+^) and granzyme K^+^ (*GZMK*^+^) CD8^+^ T cells, which have been previously characterized in the synovium of patients with RA (11). We also identified a distinct CD8^+^ T cell population, tissue-resident memory T cells (TRM), marked by expression of *ZNF683*, which encodes HOBIT (12). IFN stimulated CD8^+^ memory T cells were enriched with IFN-stimulated genes such as *IFIT1* and *IFIT3*. CD4^+^ T cells consisted of *CCR7*^+^*TCF7*^+^ naive CD4^+^ T cells, *FOXP3*^+^ Tregs (13), and an *IFNG*^+^*TBX21*^+^ population designated as type 1 helper T cell (Th1). We also identified CD4^+^ T cells expressing *CXCL13* and *IL21*, corresponding to peripheral T cells and follicular T cells (T_PH_/T_FH_) (14). We further detected *TRDC*^+^ γδ T cells and innate-like T cells expressing *ZBTB16* (encoding PLZF) and NK receptor *KLRB1*, with transcriptional profile resembling invariant NK T cells (iNKT cells) (15) and mucosal associated innate T (MAIT) cells (16). Innate subpopulations, NK cells, and ILCs were also identified. ILCs were characterized by *NCAM1* and *CD3E* negativity and enrichment for *IL5* and *IL17A*, suggesting the presence of ILC2 and ILC3 subset (17).

B cell–plasma cells comprised *MS4A1*^+^ (encoding CD20) B cells, *CD38*^+^ plasma cells, and *MKI67*^+^ plasmablast populations (Supplemental Figure 1E).

Myeloid cells encompassed diverse macrophage populations, DCs, plasmacytoid DCs (pDC), and mast cells (Supplemental Figure 1F). The macrophage clusters comprised several distinct clusters: *MERTK*^+^ macrophages, characterized by the enrichment of scavenger receptor *CD163* and *MRC1* (encoding CD206) and *MERTK* (18); *TREM2*^+^ macrophages, defined by high expression of *TREM2* as well as tissue-remodeling chemokines such as *MMP9* (19); proinflammatory macrophages expressing mediators such as *CXCL9*, *CXCL10*, and *IL1B*; and *CCR2*, a chemokine receptor indicative of infiltrating monocyte-derived macrophages (20–22). We also identified a subset of stressed macrophages marked by high expression of endoplasmic reticulum stress-related genes (*HYOU1*, *PDIA4*, *DDIT3*) (23). The DC cluster included *CLEC9A*^+^*XCR1*^+^*THBD*^+^ DC1 and *CD1C*^+^*CLEC10A*^+^ DC2 (24). Additionally, 1 DC cluster was characterized with *LAMP3*^+^, *CCR7*^+^, and immunomodulatory molecules such as *CD274* (encoding PD-L1), corresponding to recently described mature DCs enriched in regulatory molecules (mregDCs) (25).

Stromal tissue–associated cell types included lining and sublining fibroblasts as well as endothelial cells (Supplemental Figure 1G). Fibroblasts were classified into *PRG4*^+^ lining, *THY1*^+^ sublining fibroblasts and a mixture of *NOTCH3*^+^ sublining fibroblast and *MCAM*^+^ (encoding CD146) mural cells. Lining fibroblasts were further divided based on the expression of *DKK3*, which could play role in repair pathways (26), into *DKK3^lo^* and *DKK3^hi^PRG4*^+^ subsets and intermediate populations, which exhibited gene expression patterns between lining-sublining fibroblasts (27, 28), including *RSPO3*^+^, which could modulate Wnt/β-catenin signaling and have crucial effects on angiogenesis (29, 30). Sublining fibroblasts were further categorized based on the expression of *CXCL12*, *CD34, DKK3,* and *POSTN*. Endothelial cells were classified into *PROX1*^+^*FLT4*^+^ lymphatic and blood vascular vessels, the latter were further divided into *LIFR*^+^*SELE*^+^ venules and *NOTCH4*^+^ arteriolar endothelial cells.

To investigate the cellular sources of cytokines and receptors relevant to synovial inflammation, we examined the expression of genes from a curated cytokine signaling gene set (31) (Supplemental Figure 1H). Most cytokines and receptors showed dominant expression within specific cell populations; for instance, fibroblasts and B cells predominated among cells with detectable *IL6*, whereas *IL18* expression was primarily restricted to macrophages and DCs.

To quantitatively characterize morphological differences across cell types, we computed cell area and circularity (Supplemental Figure 2C). Stromal cells such as fibroblasts and endothelial subsets exhibited larger and more variable cell areas compared with immune cells, whereas lymphocyte populations demonstrated higher circularity, consistent with their small, rounded morphology.

### Disease-linked stromal cell population.

**Figure 2:**
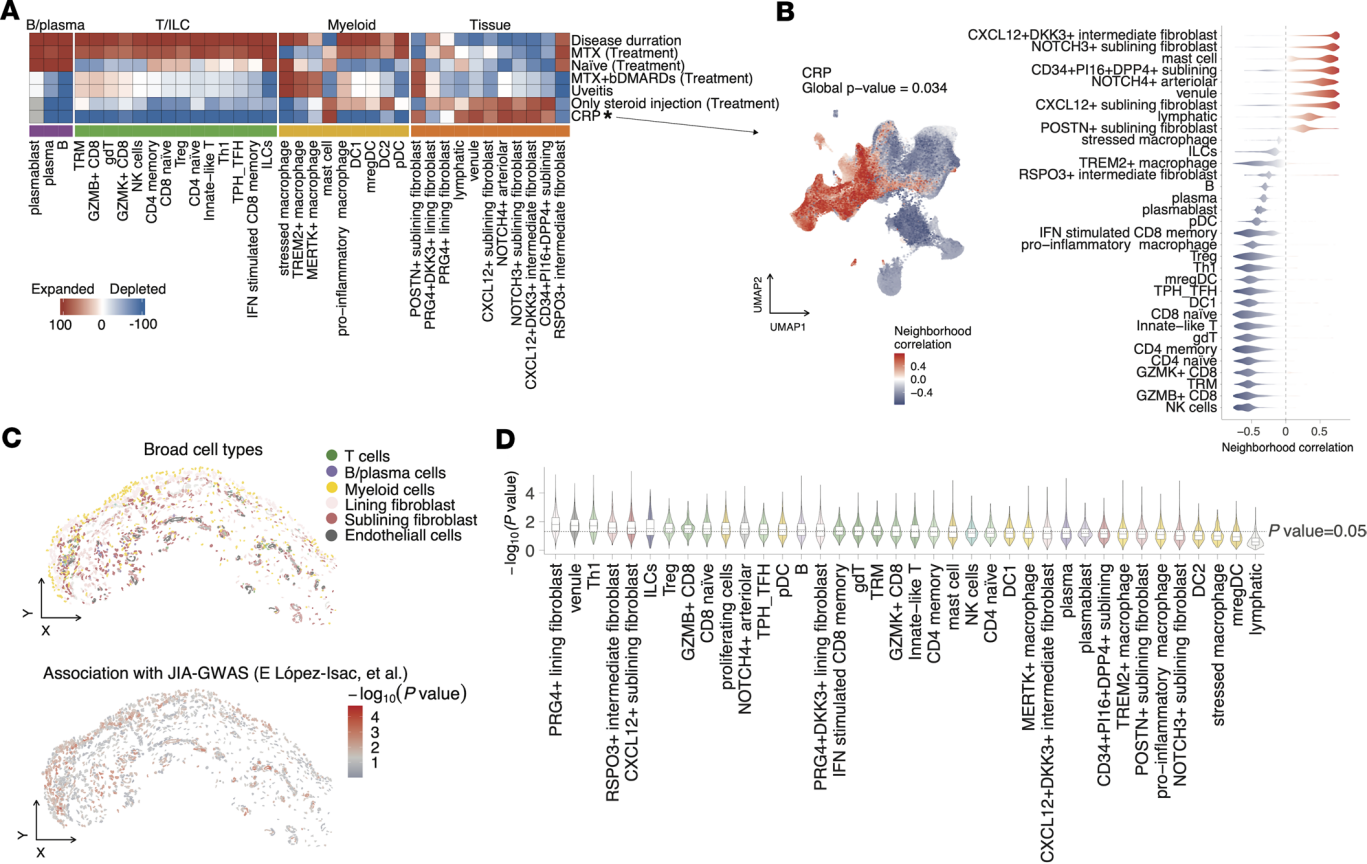
Links between synovial cell states and clinical phenotypes in JIA. (**A**) Heatmap of covarying neighborhood analysis (CNA) associations of specific cell states with each clinical variable. All testing was adjusted for age and sex. Colors represent the percentage of cell neighborhoods from each cell state with positive phenotype correlations from white to red (expanded) or blue (depleted). Clinical variables associated globally (permutation *P* < 0.05) are annotated by asterisk. (**B**) CNA for CRP association with cell states. Cells in UMAP are colored for expansion (red) or depletion (blue). (**C**) Representative region, with cells colored by broad cell types (top) and the significance of the association (−log_10_[P value]) with JIA derived from gsMap analysis (bottom). (**D**) Violin plot showing the distribution of disease associations for each spatial spot across annotated synovial cell type, ordered from left to right by decreasing median association value.

Quantitative assessment across individual biopsy samples revealed variability in cellular composition, with significant interpatient heterogeneity observed in the proportions of immune and stromal cells (Figure 1, D–G, and Supplemental Figure 2D). Correlating this heterogeneity with clinical inflammatory state using covarying neighborhood analysis (32), we found that patients with higher inflammation state defined by serum CRP levels exhibited enrichment of sublining/intermediate fibroblast and endothelial cell (venules and arteriolar endothelium) populations relative to total synovial cells, indicating a potential critical role for these cells in potentiating inflammation in JIA synovium (Figure 2, A and B), as sublining fibroblasts are known as a resource of inflammatory molecules in RA-synovium (28, 33). Conversely, some key immune populations, such as CD8^+^ T cells and proinflammatory macrophages, appeared relatively depleted in this analysis. This observation likely reflects a proportional shift rather than a decrease in absolute cell numbers; in a highly inflamed synovium, the substantial expansion of the stromal and endothelial framework can diminish the relative proportion of even an expanded immune cell compartment.

To investigate the spatial genetic relevance of cell populations within the synovium, we applied gsMap (34), a recently developed statistical framework that integrates spatial transcriptomic data with GWAS through stratified linkage disequilibrium (LD) score regression (sLDSC). This approach enables the mapping of disease heritability enrichment onto specific spatially resolved cell populations based on GWAS summary statistics of JIA (35). Notably, stromal populations — not only intermediate/sublining fibroblasts and vascular endothelial subsets, but also lining fibroblast and T cell subsets — exhibited significant enrichment of JIA-associated genetic signals (Figure 2, C and D). This suggests that these spatially localized niches — in particular, stromal cells — may contribute to not only inflammatory activity but also disease susceptibility via genetically driven mechanisms.

Together, these results offer a detailed characterization of the spatially resolved cellular landscape of JIA synovium and their association with clinical indicators and disease susceptibility.

### Spatially resolved niches reveal distinct immune-stromal compositions and gene programs.

**Figure 3:**
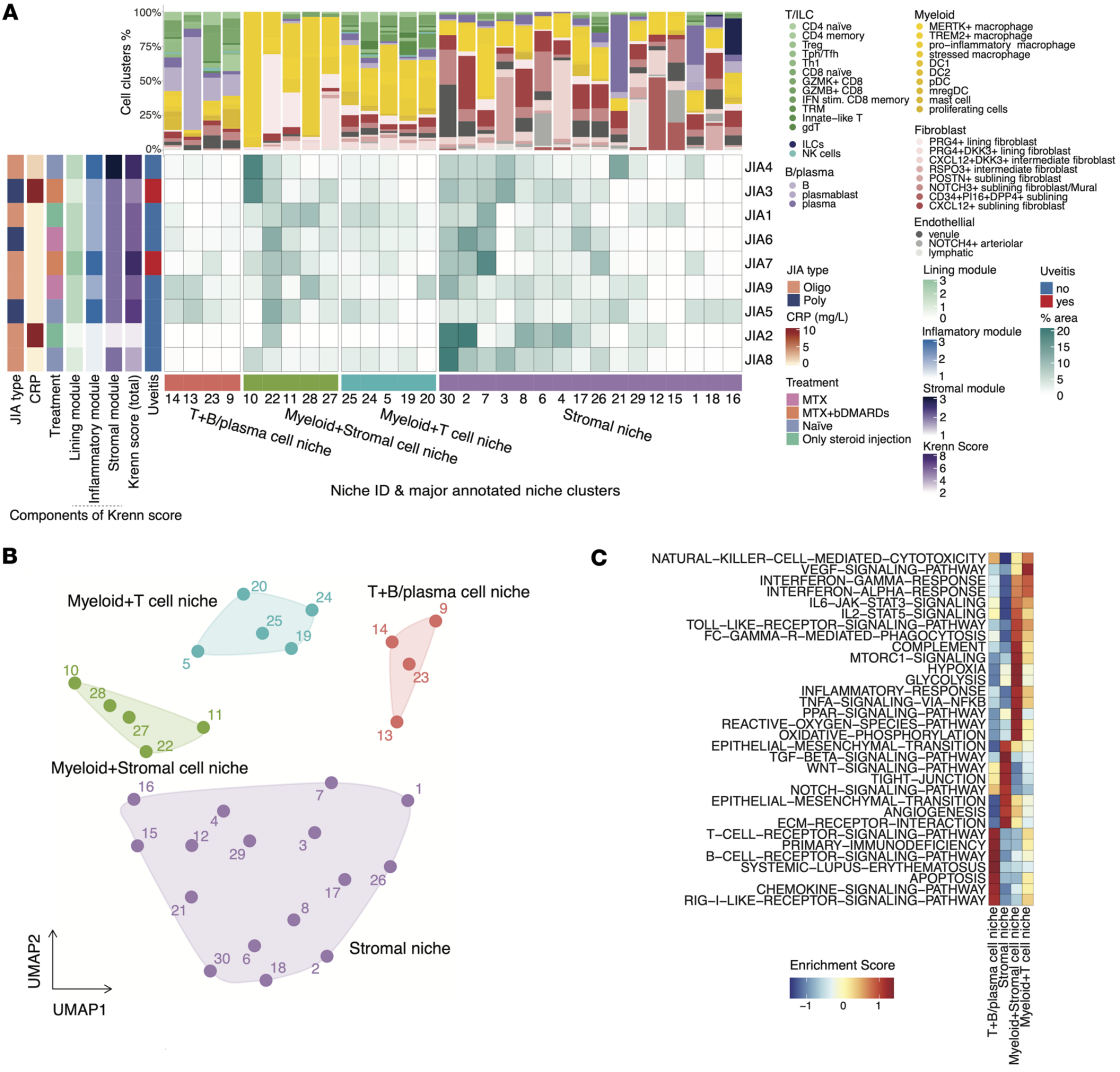
Characterization of spatial niches in JIA synovium. (**A**) The central heatmap displays the percentage area of 30 spatial niches (columns) for each patient (rows). Patient samples are annotated by clinical features on the left. The bar plots above illustrate the proportional composition of cell clusters for each corresponding niche. Spatial niches are grouped by the major annotated niche clusters shown at the bottom. (**B**) UMAP embedding of niches based on their immune-stromal cell composition. Each dot represents a niche and is colored by an annotated niche cluster. (**C**) Heatmap showing normalized gene set enrichment scores for related pathways across the 4 annotated niche classes.

To identify tissue microenvironments with distinct immune and stromal compositions, we defined spatial “niches” based on local cooccurrence patterns of cell clusters within the synovium. A spatial “niche” was defined here for each cell by examining the composition of its 30 nearest spatial neighbors, summarizing the local abundance of each cell type. Cells with similar neighborhood compositions were grouped using k-means clustering to delineate discrete spatial niches. Each niche was then annotated by its relative enrichment in major cell lineages, resulting in 4 biologically interpretable niche classes: T+B/plasma cell niche, Myeloid+Stromal cell niche, Myeloid+T cell niche, and Stromal niche (Figure 3, A and B, and Supplemental Figure 3, A and B). Analysis of these niche classes revealed the substantial heterogeneity of the synovial microenvironment across patients (Figure 3A). Even within a major class, functional diversity was observed. For example, the Myeloid+Stromal cell niche contained functionally diverse subniches: some, like Niche 10 in patient JIA3, were enriched in macrophages and corresponded with high systemic inflammation, while others were dominated by *PRG4*^+^ lining fibroblasts and exhibited a less inflammatory profile (e.g., Niche 22). This intraniche and interpatient heterogeneity highlights a granular spatial organization where specialized microenvironments can differ between individuals, even those with similar clinical classifications such as elevated CRP (e.g., JIA2 vs. JIA3).

To investigate the functional states of these niches, we performed pathway enrichment analysis comparing niche clusters. This analysis revealed class-dominant pathway signatures, including IFN-α/γ signaling in Myeloid+T cell–enriched niches, inflammatory and phagocytosis-related signatures in Myeloid+Stromal–rich niches, T and B cell receptor signaling in B+T cell–enriched niches, and angiogenesis-related pathways in stromal niches (Figure 3C). In addition, several other signaling cascades known to be implicated in JIA pathogenesis were selectively enriched across spatial niches. For instance, the IL-6/JAK/STAT3 pathways — critical in T cell differentiation (36) and chronic inflammation (37) — as well as complement components, were elevated in Myeloid+Stromal cell niches. The activation of the classical complement pathway and its association with markers of disease activity, such as elevated levels of complement-bound circulating immune complexes, have been well documented in JIA (38). Meanwhile, TGF-β and WNT signaling pathways, implicated in fibroblast activation and tissue fibrosis (39, 40), and fibroblast-mediated inflammation and treatment-refractory in RA (41, 42), were more pronounced in stromal niches.

**Figure 4:**
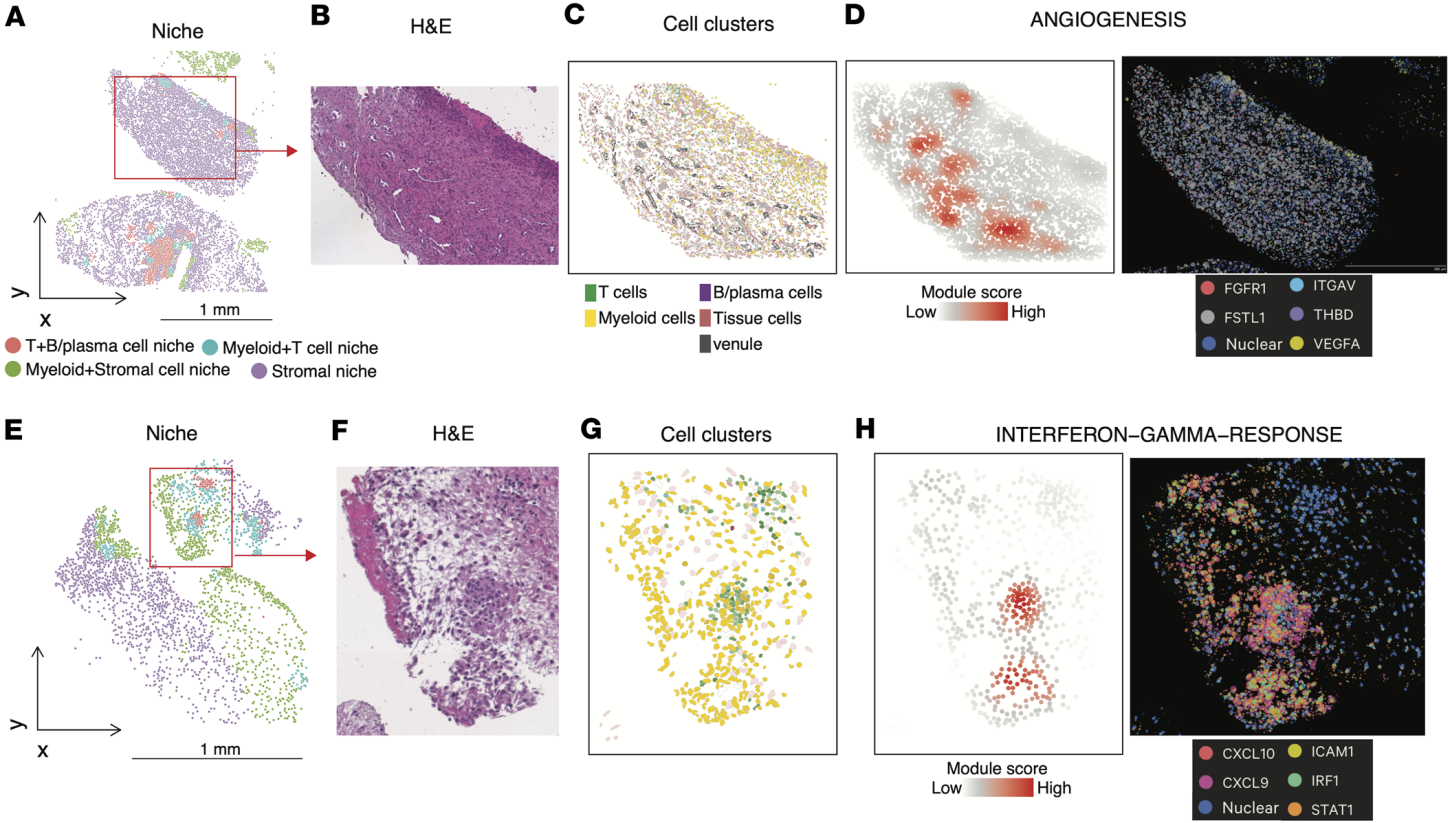
Spatial niches and associated pathway activities. (**A**–**D**) Representative images of association between niche clusters and ANGIOGENESIS pathway. Spatial mapping of annotated niches (**A**) from a representative synovial tissue section and H&E staining (**B**). (**C**) Spatial distribution of cell clusters in the zoom-in of the section in **A**. (**D**) Left, spatial enrichment score of the ANGIOGENESIS pathway. Right, Corresponding DAPI and expression of representative genes of the pathway. (**E**–**H**) Representative images of association between niche clusters and IFN-γ response pathway. Spatial mapping of annotated niches (**E**) from a representative synovial tissue section and H&E staining (**F**). (**G**) Spatial distribution of cell clusters in the zoom-in of the section in **E**. (**H**) Left, spatial enrichment score of the IFN-γ response pathway. Right, Corresponding DAPI and expression of representative genes of the pathway. Scale bars: 1 mm (**A** and **E**).

Histological mapping of niches and individual cell clusters demonstrated their anatomical relevance. For instance, stromal niches exhibited localized enrichment of the angiogenesis pathway, as visualized by spatial gene set scores (Figure 4, A–D). Another histological section also showed enrichment of IFN-γ signaling in Myeloid-T cell and Myeloid+Stromal–rich niches (Figure 4, E–H).

These findings highlight the spatial heterogeneity of the synovial immune microenvironment in JIA and demonstrate that spatial niches are associated with distinct cell type compositions and molecular programs.

### Spatial neighborhood enrichment analysis reveals NOTCH3-mediated interactions between endothelial cells and sublining fibroblasts in JIA synovium analogous to RA.

**Figure 5:**
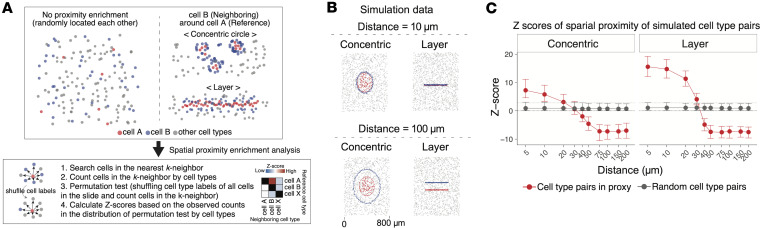
Spatial neighborhood enrichment analysis framework. (**A**) Schematic description of the spatial neighborhood enrichment analysis pipeline. The procedure includes identifying nearest neighbors, quantifying neighborhood cell type composition, performing permutation tests by shuffling cell type labels, and calculating enrichment *z* scores. (**B**) Examples of 2 simulation patterns (concentric circles and layered arrangement) utilized to evaluate the performance of the spatial neighborhood enrichment method. (**C**) Results from the simulation experiments. The *x* axis represents prespecified actual proximity distances, and the *y* axis shows calculated spatial enrichment *z* scores. The red line indicates *z* scores for cell types that were designed to be spatially close, whereas the gray line represents randomly distributed cell types. The gray dashed line indicates the multiple testing-adjusted significance threshold for *z* scores.

Since tissue-microenvironment shapes both the cell identity and localization, we aimed to characterize the cell-cell interaction in each niche based on spatial proximity. To achieve this, we developed a custom pipeline to statistically test the spatial proximity between these cell types (Figure 5A). We tested the performance of the pipeline using simulation data, assuming proximity pattern as concentric circle and layer, and found that it could efficiently identify pairs of cell types that were close to each other within 30 μm (Figure 5, B and C). To validate our spatial neighborhood analysis pipeline, we further utilized publicly available spatial transcriptomic data of human breast cancer and mouse brain (Supplemental Figure 4, A–D). Spatial neighborhood analysis revealed significant enrichment in proximity such as between cancer-associated fibroblasts (CAFs), cytotoxic T cells, astrocytes, and oligodendrocytes, underscoring their spatial interplay in the tissue architecture as described in the published study (43, 44). The human breast cancer and mouse brain datasets comprised 8,273 and 17,215 cells, respectively, and the analysis, using 4 parallel threads, was completed in 7.6 seconds and 31.7 seconds, respectively, demonstrating the computational efficiency of the pipeline.

**Figure 6:**
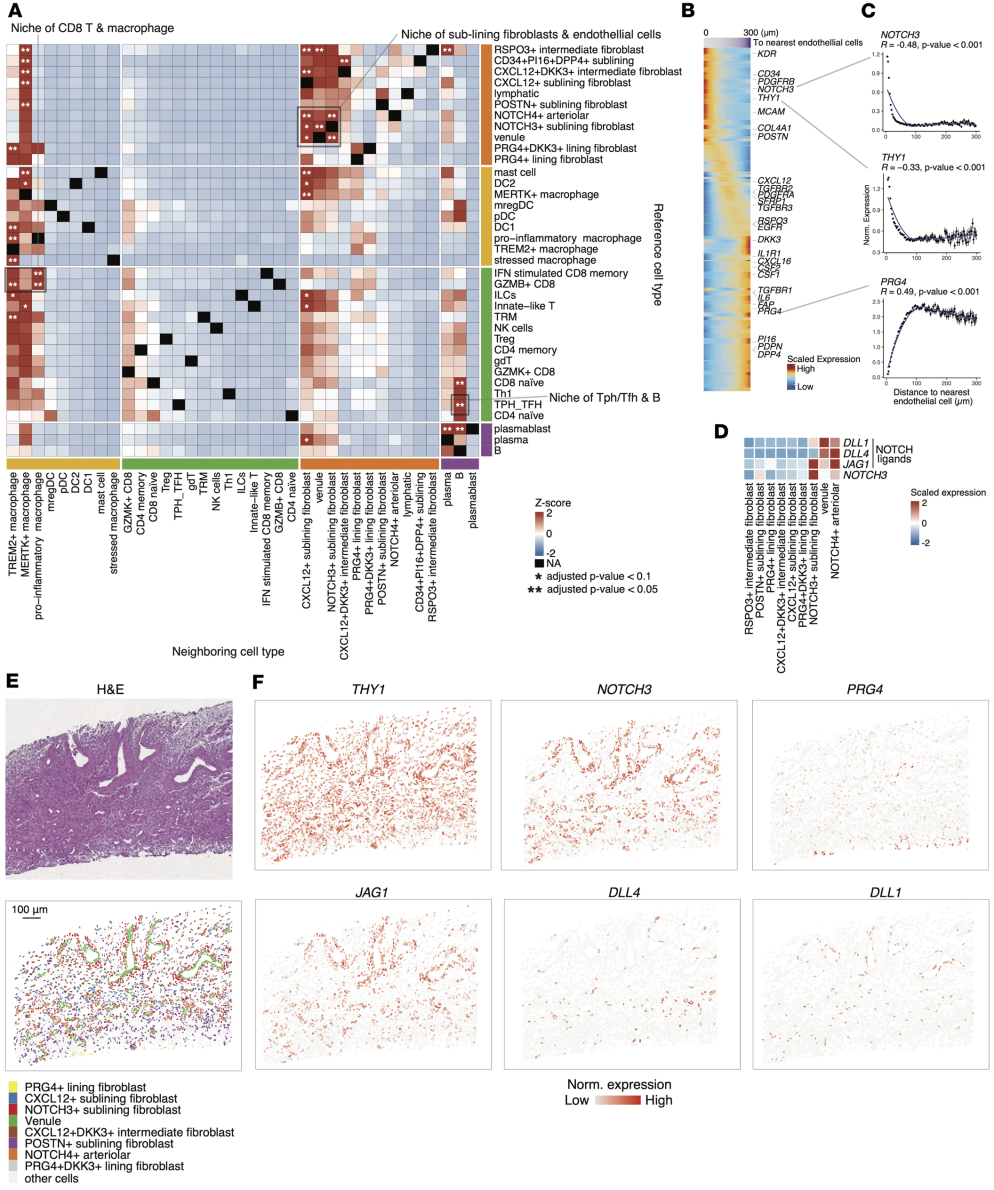
Spatial neighborhood enrichment analysis framework characterizes niche of sublining fibroblasts and endothelial cells in JIA synovium. (**A**) Spatial neighborhood enrichment using our data from samples of JIA synovium. Statistical significance is indicated as *P_adj_ < 0.1 and **P_adj_ < 0.05. The highlighted gray square indicates the niche of sublining fibroblasts and endothelial cells. *P* values were adjusted by the Benjamini-Hochberg method. (**B**) Characterization of the identified niche of sublining fibroblasts and endothelial cells. The heatmap displays the relationship between distances to nearest endothelial cells (purple bar above) and fibroblast gene expression. Rows represent fibroblast-associated genes, and columns represent individual cells. Fibroblast marker genes are labeled. (**C**) Line plots depicting binned distance to nearest endothelial cells (*x* axis) and normalized gene expression (*y* axis) of representative fibroblast genes. Statistical significance was assessed using Spearman correlation coefficients and *P* values. (**D**) Heatmap of gene expressions related to NOTCH signaling pathway components across different cell types. (**E**) Representative spatial coordinates of an identified niche of sublining fibroblasts and endothelial cells, matching pathological images. Cells are colored according to their cluster assignments. (**F**) Spatial expression patterns of fibroblast-related and NOTCH signaling-related genes within the identified niche location. Scale bar: 100 μm.

We applied this spatial neighborhood enrichment analysis pipeline to our JIA synovium spatial transcriptomic data and identified significant spatial proximities such as those between endothelial cells and sublining fibroblasts (Figure 6A). Given previous findings that endothelial cells and fibroblasts interact via NOTCH3 signaling in RA synovium (8), we further investigated whether a similar interaction exists in JIA. Indeed, we observed that *THY1*^+^ sublining fibroblasts were enriched in proximity to endothelial cells and simultaneously exhibited elevated *NOTCH3* expression (Figure 6, B and C). Consistently, endothelial cells highly expressed the NOTCH3 ligands, such as *DLL1/4* and *JAG1* (Figure 6D). Representative regions highlighting the spatial niche established by these interacting cell populations demonstrated the colocalization and reciprocal signaling between endothelial cells and sublining fibroblasts (Figure 6, E and F, and Supplemental Figure 4, E and F). This observation suggests an organized microanatomical structure within the synovial tissue, reinforcing the notion that endothelial-fibroblast interactions mediated through NOTCH3 signaling might contribute to the formation and maintenance of inflammatory niches in JIA synovium.

### Spatial crosstalk with T cells and vasculature orchestrates macrophage polarization.

**Figure 7:**
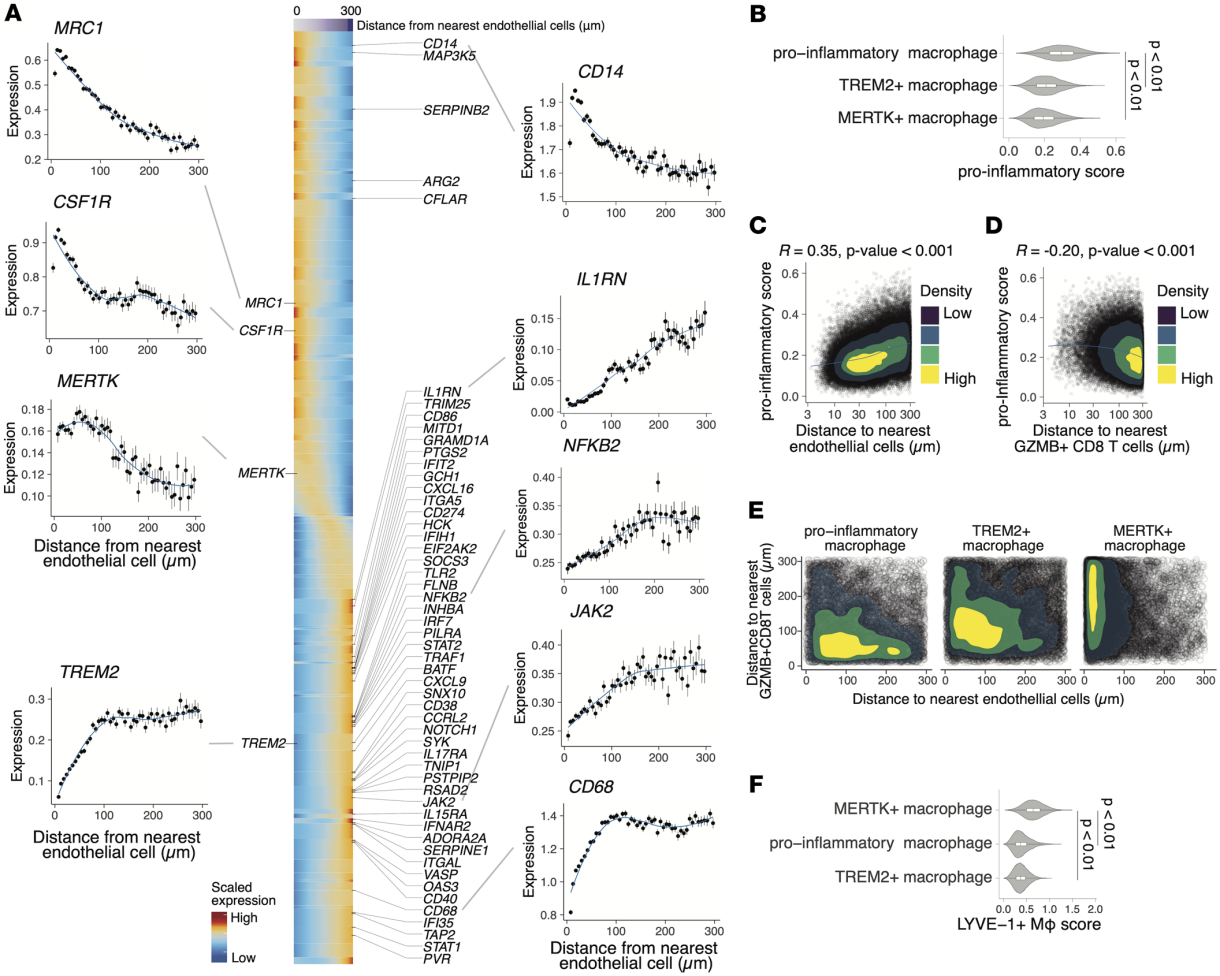
Spatial distribution of distinct macrophage states in JIA synovium. (**A**) Scaled gene expression of selected macrophage markers plotted as a function of distance from the nearest endothelial cell. Center heatmap shows scaled expression of macrophage-related genes in individual cells ordered by proximity to endothelial cells (0–300 μm), with representative genes annotated. (**B**) Violin plots showing the distribution of proinflammatory module scores across macrophage subtypes. *P* values were determined by the Wilcoxon rank-sum test. (**C** and **D**) Correlation between proinflammatory module scores of individual macrophage cells (each point) (*y* axis) and distance to the endothelial cells (**C**) or *GZMB*^+^ CD8 T cells (**D**) (*x* axis). Color-filled contours represent 2-dimensional kernel density estimates. Spearman’s correlation coefficient and *P* values are shown. (**E**) Density plots depict the distances of individual macrophage cells (each point) to the nearest endothelial cells (*x* axis) and *GZMB*^+^ CD8 T cells (*y* axis) by macrophage subtypes. Color-filled contours represent 2-dimensional kernel density estimates. (**F**) Violin plots showing the distribution of LYVE-1^+^ macrophage module scores across macrophage subtypes. *P* values were determined by the Wilcoxon rank-sum test.

Given the established importance of macrophages in the pathogenesis of inflammatory arthritis, we aimed to explore how macrophage gene expression is altered in relation to their spatial proximity to other cell types within the synovial tissues. Since monocytes enter through vasculature and differentiate to synovial macrophages (45–47), we first examined how gene expressions in macrophages correlated with their distance to endothelial cells (Figure 7A). Antiinflammatory or tissue-repairing markers such as *MRC1*, *CSF1R*, and *MERTK*, which were known to be expressed on healthy synovial tissue macrophages (48) and increased in synovium of patients with RA with remission states (18), showed higher expression in macrophages proximal to vasculature, whereas expression of proinflammatory-associated genes, including *IL1RN*, *NFKB2*, and *JAK2*, increased with distance from the nearest endothelial cell. We also noted that *TREM2*, a gene traditionally associated with tissue-repairing macrophage programs (49), exhibited a distinct spatial pattern compared with other antiinflammatory markers, as *TREM2* expression increased with distance from endothelial cells. *CD14* expression decreased while *CD68* expression concurrently increased with distance from endothelial cells, suggesting that circulating monocytes progressively differentiate into macrophages after entry through the endothelium as they adapt to the synovial microenvironment (45–47).

Next, we aimed to characterize the microenvironmental cues that induce inflammatory gene expression in macrophages. As expected from our definitions of cell type clusters, proinflammatory macrophages exhibited the highest proinflammatory module scores (50, 51) (Figure 7B). We observed a positive correlation between proinflammatory polarization and distance from endothelial cells (*R* = 0.35, *P* < 0.001) (Figure 7C). Since we found that proinflammatory macrophages are colocalized with *GZMB*^+^CD8^+^ T cells (Figure 6A), we asked whether inflammatory gene expression in macrophages is induced when they are in close proximity to CD8^+^ T cells. Indeed, proinflammatory scores in macrophages gradually decreased along with distance from *GZMB*^+^ CD8^+^ T cells (*R* = −0.20, *P* < 0.001) (Figure 7D), and a similar trend was observed for *GZMK*^+^CD8^+^ T cells (*R* = −0.11, *P* < 0.001) (Supplemental Figure 5A). While the strength of these spatial correlations is modest, their statistical significance is suggestive of this pathogenic interaction. Importantly, 2-dimensional density contour plots revealed distinct spatial patterns across macrophage subsets (Figure 7E); proinflammatory macrophages were located closer to *GZMB^+^* CD8^+^ T cells than endothelial cells.

In contrast, *MERTK*^+^ macrophages showed a strong spatial association with endothelial cells. To further investigate the potential identity of *MERTK*^+^ macrophages, we examined their similarity to LYVE-1^+^ perivascular macrophages, which have been previously described as collagen-remodeling cells positioned near blood vessels in steady-state tissues. As LYVE-1 itself was not included in our Xenium probe panel, we inferred LYVE-1^+^–like phenotype by computing gene module scores across macrophage clusters using a gene signature associated with LYVE-1^+^ macrophages (18). The analysis revealed that *MERTK*^+^ macrophages exhibited the highest LYVE-1^+^ macrophage score among the identified subsets (Figure 7F). This result supports the notion that *MERTK*^+^ macrophages in JIA synovium represent a transcriptionally related population to homeostatic LYVE-1^+^ perivascular macrophages, potentially involved in vascular support and extracellular matrix turnover.

Given the reciprocal interactions of macrophages and fibroblasts (52), we examined their spatial relationships with lining fibroblasts. We observed that while several macrophage subsets were located in proximity to lining fibroblasts, this was also clearly apparent for *TREM2*^+^ macrophages (<50 μm) (Supplemental Figure 5B). To further characterize *TREM2^+^* macrophage populations, we computed a module score using the CX3CR1^+^ lining macrophage gene signature previously defined in a murine model of joint inflammation (53). We observed significantly higher CX3CR1^+^ lining macrophage scores in *TREM2*^+^ macrophages, compared with other macrophage subsets (Supplemental Figure 5C). These results suggest that *TREM2*^+^ macrophages in our dataset may represent a human equivalent of CX3CR1^+^ barrier-forming macrophages, supporting their putative role in maintaining tissue homeostasis and limiting inflammatory responses at the synovial interface (53, 54).

### Colocalization score schema highlights crosstalk between T cells and macrophage.

**Figure 8:**
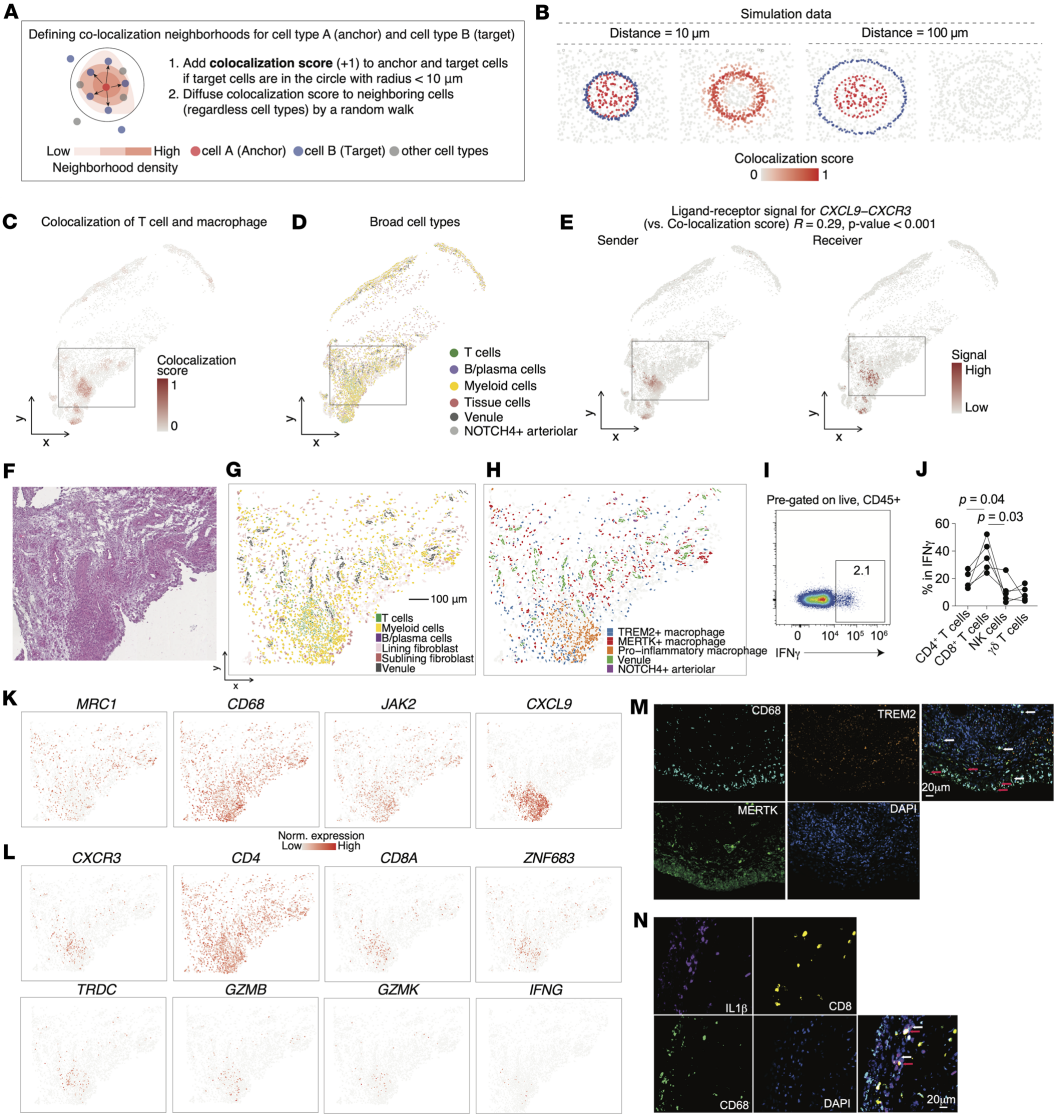
Colocalization score framework pinpoints interactions of T cells and macrophage. (**A**) Schematic illustrating spatial colocalization scoring between anchor cells and target cells within a 10 μm radius. (**B**) Representative spatial maps of high- and low-colocalization scores between anchor and target cells across tissue sections by simulated patterns. (**C**) Spatial heatmap of colocalization scores between T cells and macrophages. (**D**) Spatial map of broad cell type annotation including T cells, myeloid cells, B/plasma cells, and tissue cells. (**E**) Spatial ligand–receptor analysis results for CXCL9-CXCR3, which exhibited the strongest correlation with macrophage–T cell colocalization scores. (**F**–**H**) Zoom-in of the highlighted gray square region in **C**–**E** showing identified niche of T cells, macrophages, and endothelial cells. H&E staining image (**F**) and spatial mapping colored according to their cluster assignments (**G** and **H**). (**I**) Representative flow cytometry plot showing intracellular IFN-γ expression. (**J**) Quantification of the frequencies of CD4^+^ T cells, CD8^+^ T cells, NK cells, and γδ T cells among IFN-γ^+^ cells from the synovial fluid of oligo/poly patients with JIA (*n* = 4). Each line connects measurements from the same individual across different cell types. *P* values were determined by Friedman’s test. (**K** and **L**) Spatial expression of representative marker genes for macrophages (**K**) and T cells (**L**). Color scale indicates normalized expression levels. (**M**) Representative immunofluorescence image showing the localization of CD68^+^MERTK*^+^* macrophages (white arrows) and CD68^+^TREM2*^+^* macrophages (red arrows) in the synovial tissue. Nuclei were counterstained with DAPI (blue). (**N**) Representative immunofluorescence image demonstrating physical colocalization of CD8^+^ T cells (red arrows) and IL-1β^+^CD68^+^ proinflammatory macrophages (white arrow) within inflammatory niches of the synovium. Nuclei were counterstained with DAPI (blue). Images are representative of *n* = 5 independent samples. Scale bars: 100 μm (**G**); 20 μm (**M** and **N**).

Although our spatial neighborhood enrichment analysis effectively captured cell type interactions, it did not resolve individual cell-level proximities. To overcome this limitation and enable identification of direct cellular interactions at single-spot resolution, we developed a colocalization score framework designed to precisely capture interactions between specific cell populations within spatial transcriptomics data (Figure 8A). We implemented this new scoring method, leveraging spatial coordinates to assign higher scores to cells that coexist closely within the spatial neighborhood. Using simulated and public spatial transcriptomics data, we confirmed the robustness of this approach, demonstrating that our colocalization score accurately identified known pairs of spatially proximal cells under various controlled scenarios (Figure 8B and Supplemental Figure 5, D and E).

Subsequently, we utilized the colocalization score to investigate ligand-receptor signaling interactions between macrophages and T cells by integrating ligand-receptor pair predictions from the COMMOT pipeline (55) (Supplemental Figure 5F). The distribution of correlation *P* values showed ligand-receptor interaction signals that were spatially covaried with macrophage–T cell colocalization scores (Supplemental Figure 5G). CXCL9/CXCR3 ligand-receptor pair exhibited the highest correlation with the macrophage–T cell colocalization scores, implicating this chemokine signaling axis as a potential critical mediator facilitating spatially organized crosstalk between these immune cells within JIA synovium (Figure 8, C–E, and Supplemental Figure 5, H–J).

We next focused on the niches composed primarily of macrophages, endothelial cells and T cells (Figure 8, F–H, and Supplemental Figure 5K). While both *MERTK^+^* and *TREM2^+^* macrophages were distributed across the lining and sublining layers, these subsets exhibited partially distinct spatial patterns; *MERTK^+^* macrophages showed local enrichment around endothelial cells, whereas *TREM2^+^* macrophages were broadly present in both lining and sublining areas. This distribution of *TREM2^+^* macrophages is consistent with findings in RA, where they are observed in both compartments in the active disease state (18). On the other hand, proinflammatory macrophages were colocalized with T cells. Within the Myeloid+T cell niche, *GZMB*^+^CD8^+^ T cells exhibited the high expression of *IFNG* transcripts, which drives macrophage polarization toward a proinflammatory phenotype (Supplemental Figure 5L). We further quantified IFN-γ production by lymphocyte subsets in synovial fluid samples from oligo/poly patients with JIA, and we confirmed that CD8^+^ T cells are the predominant source of IFN-γ (Figure 8, I and J). Spatial transcriptomic mapping demonstrated enriched expression of macrophage-related inflammatory genes precisely at these niches (Figure 8, K and L, and Supplemental Figure 5M). Immunofluorescence staining of active JIA synovial samples confirmed that *MERTK^+^* macrophages exhibited focal accumulation within the sublining layer, likely around endothelial cells, whereas *TREM2^+^* macrophages dispersed in both lining and sublining layers (Figure 8M). Additionally, physical colocalization of CD8^+^ T cells with proinflammatory macrophages (Figure 8N) was observed in JIA synovial tissue, providing orthogonal validation of these inflammatory niches in situ.

Taken together, these findings underscore the spatial diversity of macrophage subsets in JIA synovium and suggest a dynamic phenotypical change in shaping the inflammatory macrophage phenotype via macrophage–T cell interactions, particularly through chemokine and cytokine signaling.

### Spatial identification and cellular characterization of TLS in JIA synovium using T and B cell colocalization scores.

**Figure 9:**
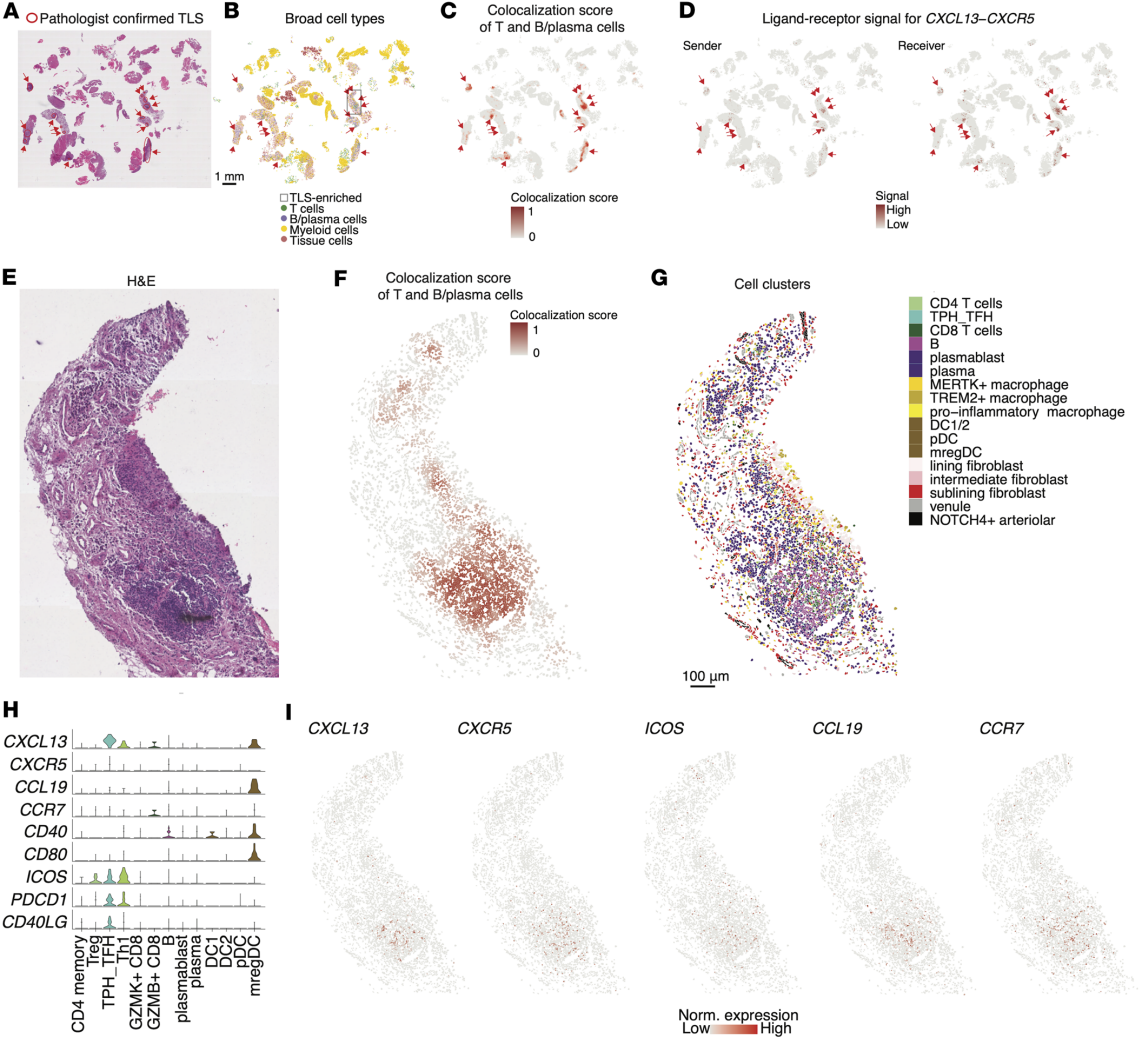
Identification and characterization of tertiary lymphoid structures (TLS) using T and B cell colocalization scores. (**A**) H&E staining images of synovial tissue, illustrating representative TLS as identified by a blinded pathologist. (**B**) Spatial distribution of T and B cells. Scale bar: 1 mm. The highlighted gray square indicates the TLS-enriched region. (**C**) Colocalization scores for T and B/plasma cells. (**D**) Spatial *CXCL13*-*CXCR5* interaction signals. (**E**) H&E staining image of zoomed-in of the area with the highest TLS density highlighted in **B**. (**F** and **G**) Same region as in **E**, colored by T and B/plasma cell colocalization score (**F**) and fine-scale cell cluster assignments (**G**). (**H**) Violin plots showing the expression levels of key TLS-related genes across different cell clusters identified within the TLS. (**I**) Spatial expression patterns of TLS-associated genes. Scale bars: 1 mm (**B**); 100 μm (**G**).

Given the established roles of TLS in chronic inflammatory conditions (56–59), we investigated their presence and cellular composition within JIA synovium. First, we applied the colocalization score framework to specifically assess interactions between T cells and B/plasma cells in order to detect potential TLS regions. These computationally identified regions matched with validated TLS regions by independent histopathological analysis performed by a blinded pathologist and T+B/plasma-rich regions by spatial mapping of broad cell types (Figure 9, A–C).

To assess the molecular mechanisms underlying TLS formation, we examined ligand-receptor signaling between T cells and B cells within identified TLS. The chemokine signaling axis CXCL13/CXCR5 showed significant enrichment in these TLS regions, suggesting a shared mechanism of TLS formation and maintenance with other chronic diseases (Figure 9D).

Histological image (Figure 9E), spatial visualization of TLS colocalization scores (Figure 9F and Supplemental Figure 6), and distribution of cell clusters confirmed the enrichment of Tph/Tfh cells within TLS-dense regions (Figure 9G). These clusters coexpressed high levels of key TLS-related genes including *CXCL13, CXCR5, ICOS,* and *CCR7* (Figure 9, H and I). Notably, we also identified mregDCs as a likely source of *CCL19*, the ligand for *CCR7*, pointing to a potential cellular axis contributing to the establishment of TLS-associated chemotactic gradients in JIA. The elevated expression of *CD40* and *CD80* in mregDCs may facilitate the priming of naive T cells.

**Figure 10:**
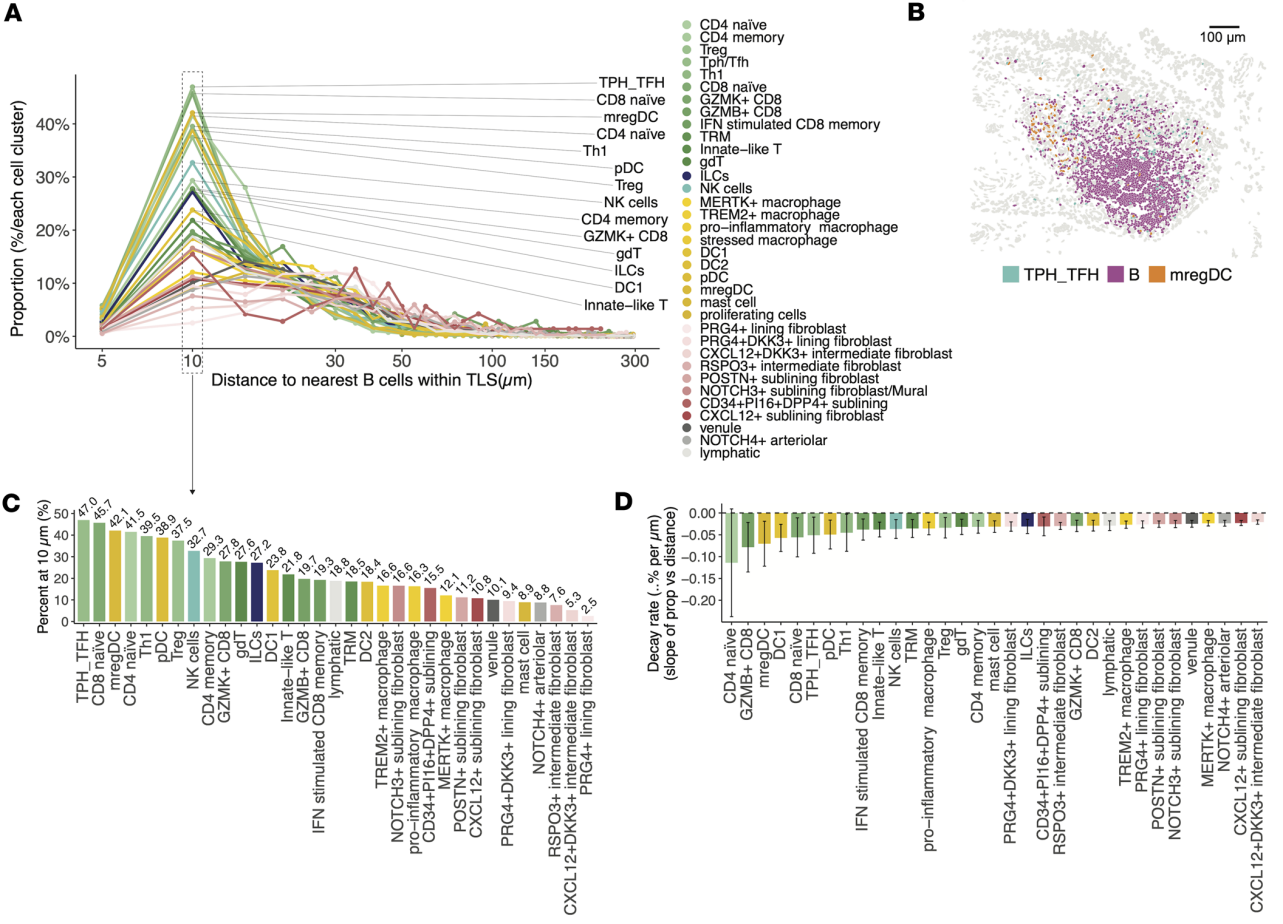
Composition of cell states in TLS. (**A**) Line plot showing the proportion of each immune cell cluster (*y* axis, percentage of total cells within each cluster) relative to its binned distance to the nearest B cell within identified TLS regions (*x* axis, log-scaled). Each line represents a distinct immune cluster, with colors corresponding to the cluster identities. Points denote the proportion per 5 μm distance bin, and annotations on the right indicate the top clusters based on peak proximity to B cells. (**B**) Representative TLS region showing colocalization of Tph/Tfh cells, B cells, and mregDCs. (**C**) Bar plot showing the percentage of each immune or stromal cell cluster located within 10 μm of a TLS B cell. Percentages are displayed above each bar. (**D**) Estimated decay rates of cell cluster proximity to TLS B cells, expressed as the slope of the proportion of cells per μm distance. Negative values indicate stronger spatial enrichment close to TLS B cells with rapid decline at increasing distances. Data are shown as 95% CI from linear regression fits.

To further characterize the spatial organization of cell types within TLS, we quantified the distance from each cell to its nearest B cell across identified TLS regions and calculated the distribution of cell types across distance. This analysis revealed that several immune cell populations, particularly T cells as expected, including Tph/Tfh cells as the most enriched cell types, proxy for B cells (Figure 10A). These spatial features highlight the coordinated positioning of TLS-associated immune and stromal populations, supporting their roles in TLS formation and maintenance, likely through CXCL13/CXCR5 (T+B) and CCL19-mediated signaling from mregDCs (Figure 10B). We summarized the percentage of each cell cluster within 10 μm of B cells and compared the rate of spatial decay with distance (Figure 10, C and D). Tph/Tfh cells and mregDCs showed high enrichment and steepest decay, indicating strong localization near B cells. Together, these findings demonstrate the utility of the colocalization score in identifying TLS in JIA synovium and provide insights into the complex cellular interactions and signaling pathways driving TLS formation and maintenance.

### Integrated comparison of JIA and RA synovial tissue reveals potential disease-specific cellular niches.

**Figure 11:**
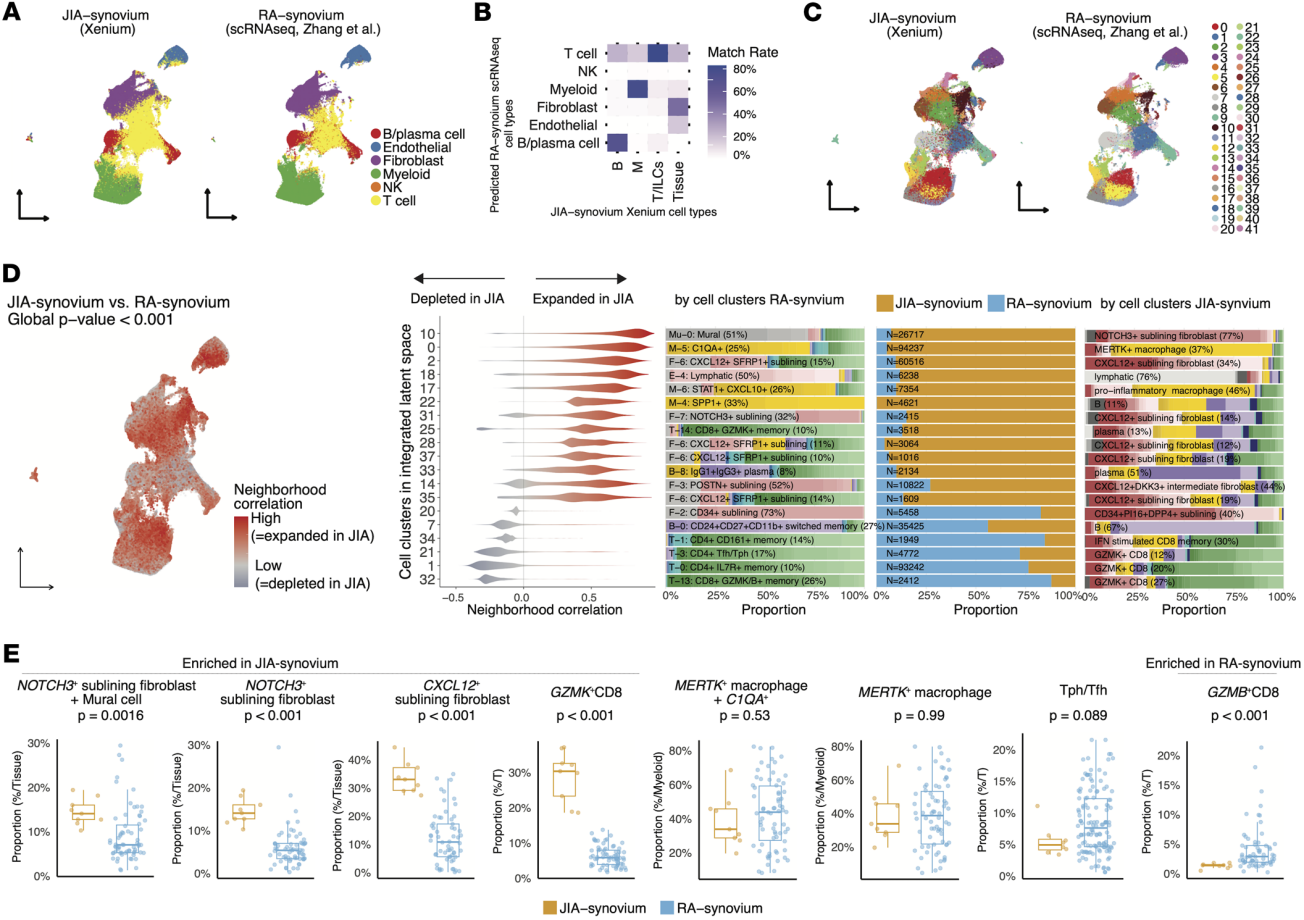
Comparative analysis of JIA and RA synovium using integrated spatial-transcriptomic and scRNA-Seq data. (**A**) Cell type level label transfer results, where RA synovium scRNA-Seq was used as the reference and JIA synovium Xenium data as the query. RA-derived annotations were transferred to individual JIA cells using a k-nearest neighbors (kNN) approach in the integrated latent space. (**B**) Match rate between transferred RA-synovium cell types and our original JIA-synovium-based annotations. Matrix shows percentage overlap between each transferred and original broad cell type. (**C**) Cell clustering in the integrated latent space. UMAP visualizations of both JIA (Xenium) and RA (scRNA-Seq) cells are colored by newly defined shared clusters. (**D**) CNA identifies integrated-space clusters that are expanded or depleted in JIA synovium relative to RA synovium. Sex and disease duration were adjusted for, while age was excluded from covariates due to its collinearity with disease status, reflecting the distinct age of onset between RA and JIA. Left: Spatial visualization of cells colored by neighborhood correlation (red, expanded in JIA; blue, depleted). Middle: Violin plots showing distribution of neighborhood correlations for each integrated cluster. Only clusters with cells either of positive or negative correlation > 80% are shown. Right: Bar plots showing cell-type composition of each cluster in RA (left) and JIA (right) synovium. For each cluster, the most abundant original cell type (from either RA or JIA) is annotated with text in each bar. Center bar plot showing relative proportion of cells derived from JIA versus RA within each cluster (orange, JIA; blue, RA). (**E**) Box plots showing the lineage-normalized proportion of cell clusters across individual samples, stratified by condition (JIA versus RA synovium). *P* values were calculated using Wilcoxon rank-sum tests. Box plots showing the median, interquartile range, and 1.5× interquartile range (IQR) whiskers.

To compare the cellular architecture of JIA and RA synovium, we integrated our spatial transcriptomic data with publicly available RA synovial scRNA-Seq data (28) (Methods). RA-derived cell clusters were transferred to JIA cells in the shared latent space (Figure 11A). The transferred cell type labels showed high concordance with our annotations for major immune and stromal compartments in JIA synovium, including T cells/ILCs, B/plasma cells, myeloid cells, and stromal tissue cells (Figure 11B), with detailed results for each lineage shown in Supplemental Figure 7.

Unsupervised clustering in the integrated latent space yielded 42 shared cell clusters encompassing cells from both JIA and RA samples (Figure 11C). To identify disease-relevant cell states, we applied a CNA approach. This approach identified clusters with significantly different neighborhood compositions between the 2 conditions; for instance, integrated-clusters 10 was expanded in JIA, while integrated-clusters 21 was depleted (Figure 11D).

To mitigate potential technical artifacts arising from differences in tissue dissociation sensitivity between spatial transcriptomics and scRNA-Seq (60), we performed lineage-normalized comparisons between JIA and RA by calculating the relative abundance of key subsets within major immune lineages, with focus on cell populations, which were dominant in skewed integrated cell clusters toward JIA or RA (Figure 11E). Specifically, *NOTCH3*^+^ sublining fibroblasts (matched with mural cells in the RA-cluster), *CXCL12*^+^ sublining fibroblast cells, and *GZMK*^+^CD8^+^ T cells were enriched in JIA, whereas clusters enriched in RA included *GZMB*^+^CD8^+^ T cells. Although it did not show statistical significance, Tph/Tfh cells showed a trend toward enrichment in RA (*P* = 0.089). Collectively, these findings demonstrate the power of integrated spatial single-cell analysis to reveal potential cellular distinctions between JIA and RA pathogenesis.

## Discussion

In this study, we present the largest subcellular-resolution spatial transcriptomic atlas, to our knowledge, of JIA synovium. We profiled whole synovial sections with single-molecule transcript detection, capturing a rich landscape of diverse immune and stromal cell populations in situ. By mapping cells in their native tissue context, our atlas reveals how innate and adaptive immune cells, fibroblasts, and endothelial cells organize into discrete pathogenic niches within the JIA joint. Key inflammatory cell states identified (e.g., proinflammatory macrophages, Tph/Tfh, *GZMK*^+^/*GZMB*^+^ CD8^+^ T cells, innate-like T cells, B/plasma cells, and distinct fibroblast subsets) spatially colocalize in patterns consistent with functional crosstalk.

Our findings corroborate the niche architecture and stromal-immune hubs as previously reported in treatment-naive JIA synovium (7). We extend that work by (a) leveraging high-plex, subcellular spatial data and a single-spot colocalization/neighborhood framework that identifies endothelial-*NOTCH3*^+^ sublining fibroblast and CXCL9/CXCR3 macrophage–CD8^+^ T cell circuits in situ; (b) linking JIA genetic risk to defined fibroblast states; and (c) quantitatively resolving TLS composition and chemokine context.

The identification of spatially resolved tissue niches within JIA synovium reveals distinct immune-stromal architectures that likely underpin localized pathogenic processes. Our unsupervised classification of microanatomical niches into 4 major classes — T+B/plasma cell, Myeloid+Stromal, Myeloid+T cell, and Stromal — reflects the complex cellular crosstalk shaping the inflamed synovial landscape. The Myeloid+T cell and T+B/plasma cell niches exhibited enrichment for IFN-α/γ signaling and adaptive immune pathways, respectively, echoing findings from adult RA studies in which Th1-type cytokines and B cell–T cell interactions dominate lymphoid-rich synovitis (28). The presence of angiogenesis-associated signatures within stromal-rich niches further supports prior work suggesting that fibroblast cells with enrichment for genes associated with the vasculogenesis program sustain chronic inflammation (61, 62). These spatially confined signaling environments may facilitate niche-specific therapeutic resistance or responsiveness, as prior study has shown that pauci-immune synovium with abundant fibroblasts is associated with poor therapeutic response in RA (28, 63). Future studies integrating spatial proteomics and longitudinal biopsy cohorts could clarify how these niches evolve over disease course or in response to therapy, ultimately advancing precision medicine strategies in JIA.

Synovial fibroblasts are broadly classified into lining and sublining fibroblasts: sublining fibroblasts exhibit a secretory phenotype characterized by high expression of inflammatory molecules such as IL-6, CXCL12, and CCL2, which recruit monocytes, whereas lining fibroblasts are the source of the metalloproteinase as the main driver of invasion and cartilage degradation (64, 65). A striking finding in our study is the spatial coupling of sublining fibroblasts with the synovial endothelium via NOTCH3 signaling, a niche interaction that mirrors observations in RA (8). In RA, endothelium-derived NOTCH signals imprint a positional identity on perivascular fibroblasts, promoting an aggressive inflammatory phenotype (8). The shared endothelial-fibroblast niche architecture in JIA and RA underscores a fundamental pathological circuit and raises the hypothesis that targeting the NOTCH3 pathway could attenuate fibroblast pathogenicity in JIA, echoing therapeutic insights previously shown in RA models (8). Interestingly, when integrating the JIA Xenium spatial transcriptomic data with RA scRNA-Seq data, many of the *NOTCH3*^+^ fibroblasts in JIA synovium were predicted to align with mural cell identities, suggesting continuous cell states between them. This finding is congruent with the previous study showing that there are no universal specific markers to distinguish fibroblasts and mural cells (66) and that mural cells can arise from perivascular fibroblast-like cells through NOTCH signaling (67). Furthermore, when we applied gsMap, *PRG4*^+^ lining fibroblasts showed the strongest association with JIA GWAS signals. This finding stands in contrast to the inflammatory sublining fibroblasts enriched in patients with high CRP and suggests that distinct fibroblast phenotypes may be differentially involved in disease susceptibility versus disease activity or progression. Such a model parallels observations in systemic lupus erythematosus (SLE), where gene programs associated with disease-state differ from those linked to severity (68).

Our spatial analysis also uncovered intimate interactions between infiltrating T cells and synovial macrophages that appear to form a positive feedback loop driving proinflammatory-polarized inflammation. In JIA synovium, *CXCR3*^+^ T cells were frequently colocalized with *CXCL9^+^CXCL10^+^* proinflammatory macrophages. CXCL9/CXCR3 or CXCL10/CXCR3 chemokine axis is known to recruit and activate Th1 cells in inflamed joints (69): synovial macrophages (and lining cells) secrete CXCR3 ligands CXCL9/CXCL10 under IFN-γ stimulation (70), which align with enrichment of IFN-α/γ signaling in Myeloid+T cell niches described in our analysis. Our data support this model in situ, suggesting that T cells and proinflammatory macrophages engage in reciprocal activation — effector T cells maintain macrophages in a proinflammatory state, and macrophage-derived CXCL9/CXCL10 attracts more T cells into the tissue. Importantly, our spatial transcriptomic data reveal that, while *GZMB*^+^CD8^+^ T cells are a rare population (representing < 2% of total T cells), they exhibit the highest per-cell expression of IFN-γ transcripts, highlighting them as a small but potent potential source of this key cytokine.

*MERTK*^+^ macrophages and *TREM2*^+^ macrophages identified in our study correspond to functional analogs of LYVE-1^+^ perivascular macrophages and CX3CR1^+^ lining macrophages previously described in mouse models, respectively (53, 71). In parallel, RA remission has been associated with *MERTK*^+^ macrophage subsets, including *LYVE1*^+^ and *TREM2*^hi^ populations (18). The spatial proximity of *MERTK*^+^ macrophages to endothelial cells, combined with their transcriptional signature, supports a role for these cells in vascular maintenance and resolution of inflammation in the pediatric synovium. In RA, *MERTK^+^LYVE1^+^* macrophages predominantly reside in the lining layer during homeostasis and remission but shift to perivascular regions in the sublining layer during active inflammation (18). The localization of *MERTK^+^* macrophages in our JIA samples mirrors this pattern and may reflect the inflammatory state of the tissue, as all biopsies in our study were obtained from clinically active joints. Moreover, recent work has highlighted a specialized vascular-immune interface at the synovial lining-sublining boundary, where fenestrated *PV1*^+^ endothelial (proxy for venules in our dataset) cells permit entry of circulating immune complexes, initiating inflammatory responses (47). These are sensed by resident macrophages and nociceptor neurons, which act as components of the synovial defense system and potential secondary effectors of fibroblasts. The enrichment of endothelial-fibroblast interactions and the anatomical proximity of perivascular macrophage subsets in JIA synovium are consistent with this emerging model of immune surveillance.

Using spatial colocalization analysis, we identified organized aggregates of B cells, T cells, and DCs in the synovium, often with structures reminiscent of germinal centers. These TLS-like aggregates in JIA closely mirror those observed in adult autoimmune diseases such as RA and SLE, amplifying local autoimmunity and inflammation (72, 73). Likewise, JIA TLS may serve as sites of local antigen presentation and lymphocyte activation, potentially driving epitope spreading or autoantibody production even in a pediatric setting, although the specific antigens involved remain unknown. In the context of JIA, lymphoid aggregates in the synovium have been reported (58). Intriguingly, a prior study by Gregorio et al. reported that these nascent TLS in JIA did not strongly associate with disease duration or severity, though they found them to be more common in antinuclear antibody^+^ (ANA^+^) cases. The TLS we observed shared molecular features with TLS in other disorders — for example, JIA TLS areas showed upregulation of lymphoid chemokines *CXCL13* from Tph/Tfh cells and *CCL19* from mregDCs, and *CD40* in B cells (74). These findings encourage further study of TLS as a prognostic marker and as a potential therapeutic target, especially given that some of the agents targeting TLS-related pathways are currently in clinical trials for RA (75).

While JIA and RA are distinct diseases (1), our findings might enable a cross-comparison of pediatric vs. adult synovial pathogenesis. Within stromal cells, the cellular composition of inflamed JIA synovium skewed toward inflammatory elements, including *NOTCH3*^+^ and *CXCL12*^+^ sublining fibroblasts compared with RA. The preferential enrichment of *GZMK^+^*CD8*^+^* T cells in JIA and *GZMB^+^*CD8^+^ T cells in RA suggests that divergent modes of tissue inflammation and immune activation via GZMK and GZMB pathways (76, 77) may contribute to different pathogenic mechanisms in pediatric versus adult arthritis. Furthermore, they may reflect differences between seronegative JIA (RF-negative) and seropositive RA, the latter of which includes the majority of patients with RA in our comparison, with over 75% testing positive for anticyclic citrullinated peptide antibodies. We therefore acknowledge that these findings are preliminary and that future studies in larger, prospectively matched cohorts are needed to confirm these cellular distinctions. Nonetheless, these contrasts between adult and pediatric arthritis, although preliminary, provide hypotheses about how age and immune maturation influence the inflammatory microenvironment.

Methodologically, this study introduces a computational pipeline for quantifying spatial colocalization at both cell-type and single-spot levels. The latter approach yielded quantitative “colocalization scores” for cell pairings and enabled linking with the established framework for inferring ligand-receptor signals. While prior single-cell studies in RA inferred cell interactions from dissociated cells (78–81), our pipeline provides a spatially resolved view of cell communications, adding confidence by requiring physical proximity in tissue. We envision that this analytic framework can be applied to other spatial omics datasets, enabling a deeper understanding of tissue organization and cross-talk in health and disease.

This work has several limitations that warrant discussion. First, the number of patient samples is modest, reflecting the difficulty of obtaining pediatric synovial biopsies; thus, the generalizability of some findings (e.g., TLS presence or specific rare cell populations) will need confirmation in larger JIA cohorts. Relatedly, while we systematically tested associations between all cell populations and multiple clinical covariates — including disease duration, treatment status (e.g., MTX, bDMARDs, steroid injection), and the presence of uveitis — we found no statistically significant associations apart from CRP levels, underscoring the need for validation in larger cohorts to better define the relationship between synovial cell composition and clinical phenotypes. Second, our comparisons between JIA and RA relied on integrating our spatial transcriptomics data with published scRNA-Seq data from adult RA, and we acknowledge that such cross-study comparisons are inherently indirect due to differences in tissue processing and assay platforms. However, these differences can also offer complementary insights. For instance, spatial transcriptomics has a higher capture rate of stromal cells compared with dissociative scRNA-Seq, which often underrepresents these cell types (60). This allowed us to characterize immune-stromal interactions more comprehensively in JIA synovium.

In conclusion, our high-resolution spatial transcriptomic data of oligo/poly JIA synovium in combination with potentially new computational pipelines charted the inflammatory cell atlas and microanatomy of the disease with unprecedented detail, paving the way for future studies to leverage spatial omics in understanding tissue-level immunology. By combining spatial genomics with multi-omics and experimental validation, we move closer to unraveling the cellular choreography of arthritis and translating these insights into targeted therapies for children and adults suffering from joint inflammation.

## Methods

Supplemental Methods are available online with this article.

### Sex as a biological variable.

This study considered sex as a biological variable and included both female and male patients. The patient cohort, detailed in Supplemental Table 1, consists of a higher proportion of female patients, which is representative of the known sex predominance in the epidemiology of JIA. To account for potential confounding effects, sex was included as a covariate in relevant statistical models.

### Subject recruitment and clinical data collection.

Children who met the ILAR classification criteria for a diagnosis of JIA (3) and were seen at Children’s Hospital Colorado were recruited for the study. Clinical information was obtained through a review of medical records. ANA and RF were considered positive if detected at any titer and at any point during the disease course. CRP levels were measured within 3 months of the procedure.

### Statistics.

The statistical tests performed are indicated in the figure legends or Methods. For the analysis of our data, the Wilcoxon rank-sum test was used for comparing continuous variables between 2 groups. Spearman’s rank correlation was utilized to examine the relationship between 2 continuous variables. We corrected for multiple comparisons using the Benjamini-Hochberg procedure to control the false discovery rate. Adjusted *p* values less than 0.05 were considered significant.

### Study approval.

This study was approved by the Institutional Review Board at University of Colorado School of Medicine (protocol number 23-2268). The design and conduct of this study fully complied with all relevant regulations regarding the use of human study participants, including the U.S. Department of Health and Human Services regulations for the protection of human subjects (45 CFR 46), and adhered to the ethical principles outlined in the Declaration of Helsinki.

### Data availability.

All raw and processed data will be available at dbGAP (accession no. phs004370.v1.p1). Spatial transcriptome data for benchmarking spatial neighborhood analysis and colocalization score was downloaded from https://www.10xgenomics.com/jp/support/software/xenium-onboard-analysis/latest/resources/xenium-example-data We used publicly available software for the analyses. Newly developed R package for spatial neighborhood enrichment analysis and colocalization scoring system and vignette using simulation data and public 10X spatial transcriptome data will be available on Github (https://github.com/juninamo/spatialCooccur; commit ID 6039663). Other analytic codes to generate figures are available on Github (https://github.com/juninamo/JIA_Xenium; commit 71010db). Values for all data points in graphs are reported in the Supporting Data Values file.

## Author contributions

KY, RF, NR, HL, KH, and CL recruited patients, obtained samples, and curated clinical data. RF, CL, and KH contributed to the procurement and processing of samples and study design. AHJ provided disease immunology inputs. MRC performed pathological evaluation of histological images. JI led the computational and statistical analyses. JI and KY interpreted the results, wrote the manuscript. All authors participated in editing the final manuscript.

## Funding support

This work is the result of NIH funding in part and is subject to the NIH Public Access Policy. Through acceptance of this federal funding, the NIH has been given a right to make the work publicly available in PubMed Central.

- Uehara Memorial Foundation Postdoctoral Fellowship.
- Grant-in-Aid for Japan Society for the Promotion of Science Overseas Research Fellows.
- Mochida Memorial Foundation for Medical and Pharmaceutical Research (to JI).
- NIH (K08DK128544 and R03DK144245 to KY).

## Supplementary Material

Supplemental data

Supplemental table 2

Supporting data values

## Version 1 11/21/2025

In-Press Preview

## Version 2 01/09/2026

Electronic publication

## References

[1] Martini A (2022). Juvenile idiopathic arthritis. Nat Rev Dis Primers.

[2] Thierry S (2014). Prevalence and incidence of juvenile idiopathic arthritis: a systematic review. Joint Bone Spine.

[3] Petty RE (2004). International league of associations for rheumatology classification of juvenile idiopathic arthritis: second revision, Edmonton, 2001. J Rheumatol.

[4] Oen KG, Cheang M (1996). Epidemiology of chronic arthritis in childhood. Semin Arthritis Rheum.

[5] Glerup M (2022). Changing patterns in treatment, remission status, and categories in a long-term nordic cohort study of juvenile idiopathic arthritis. Arthritis Care Res (Hoboken).

[6] Guzman J (2015). The outcomes of juvenile idiopathic arthritis in children managed with contemporary treatments: results from the ReACCh-Out cohort. Ann Rheum Dis.

[7] Bolton C (2025). Synovial tissue atlas in juvenile idiopathic arthritis reveals pathogenic niches associated with disease severity. Sci Transl Med.

[8] Wei K (2020). Notch signalling drives synovial fibroblast identity and arthritis pathology. Nature.

[9] Raab A (2021). Outcome of children with oligoarticular juvenile idiopathic arthritis compared with polyarthritis on methotrexate- data of the German BIKER registry. Pediatr Rheumatol Online J.

[10] Jeelani W (2022). The importance of differentiating oligoarticular juvenile idiopathic arthritis from lyme arthritis in pediatric patients. Cureus.

[11] Jonsson AH (2022). Granzyme K^+^ CD8 T cells form a core population in inflamed human tissue. Sci Transl Med.

[12] Mackay LK (2016). Hobit and Blimp1 instruct a universal transcriptional program of tissue residency in lymphocytes. Science.

[13] Wing JB (2019). Human FOXP3^+^ regulatory T cell heterogeneity and function in autoimmunity and cancer. Immunity.

[14] Rao DA (2017). Pathologically expanded peripheral T helper cell subset drives B cells in rheumatoid arthritis. Nature.

[15] Kovalovsky D (2008). The BTB-zinc finger transcriptional regulator PLZF controls the development of invariant natural killer T cell effector functions. Nat Immunol.

[16] Garner LC (2023). Single-cell analysis of human MAIT cell transcriptional, functional and clonal diversity. Nat Immunol.

[17] Spits H (2013). Innate lymphoid cells--a proposal for uniform nomenclature. Nat Rev Immunol.

[18] Alivernini S (2020). Distinct synovial tissue macrophage subsets regulate inflammation and remission in rheumatoid arthritis. Nat Med.

[19] Do TH (2022). TREM2 macrophages induced by human lipids drive inflammation in acne lesions. Sci Immunol.

[20] Tsou C-L (2007). Critical roles for CCR2 and MCP-3 in monocyte mobilization from bone marrow and recruitment to inflammatory sites. J Clin Invest.

[21] Palframan RT (2001). Inflammatory chemokine transport and presentation in HEV: a remote control mechanism for monocyte recruitment to lymph nodes in inflamed tissues. J Exp Med.

[22] Jakubzick CV (2017). Monocyte differentiation and antigen-presenting functions. Nat Rev Immunol.

[23] Shan B (2017). The metabolic ER stress sensor IRE1α suppresses alternative activation of macrophages and impairs energy expenditure in obesity. Nat Immunol.

[24] Collin M, Bigley V (2018). Human dendritic cell subsets: an update. Immunology.

[25] Maier B (2020). A conserved dendritic-cell regulatory program limits antitumour immunity. Nature.

[26] Kemble S, Croft AP (2021). Critical role of synovial tissue-resident macrophage and fibroblast subsets in the persistence of joint inflammation. Front Immunol.

[27] Greicius G (2018). PDGFRα^+^ pericryptal stromal cells are the critical source of Wnts and RSPO3 for murine intestinal stem cells in vivo. Proc Natl Acad Sci U S A.

[28] Zhang F (2023). Deconstruction of rheumatoid arthritis synovium defines inflammatory subtypes. Nature.

[29] Kazanskaya O (2008). The Wnt signaling regulator R-spondin 3 promotes angioblast and vascular development. Development.

[30] He Z (2023). R-spondin family biology and emerging linkages to cancer. Ann Med.

[31] Kanehisa M (2023). KEGG for taxonomy-based analysis of pathways and genomes. Nucleic Acids Res.

[32] Reshef YA (2022). Co-varying neighborhood analysis identifies cell populations associated with phenotypes of interest from single-cell transcriptomics. Nat Biotechnol.

[33] Zhang F (2019). Defining inflammatory cell states in rheumatoid arthritis joint synovial tissues by integrating single-cell transcriptomics and mass cytometry. Nat Immunol.

[34] Song L (2025). Spatially resolved mapping of cells associated with human complex traits. Nature.

[35] López-Isac E (2021). Combined genetic analysis of juvenile idiopathic arthritis clinical subtypes identifies novel risk loci, target genes and key regulatory mechanisms. Ann Rheum Dis.

[36] Eddahri F (2009). Interleukin-6/STAT3 signaling regulates the ability of naive T cells to acquire B-cell help capacities. Blood.

[37] Hirano T (2021). IL-6 in inflammation, autoimmunity and cancer. Int Immunol.

[38] Gilliam BE (2011). Significance of complement components C1q and C4 bound to circulating immune complexes in juvenile idiopathic arthritis: support for classical complement pathway activation. Clin Exp Rheumatol.

[39] Akhmetshina A (2012). Activation of canonical Wnt signalling is required for TGF-β-mediated fibrosis. Nat Commun.

[40] Biernacka A (2011). TGF-β signaling in fibrosis. Growth Factors.

[43] Broz MT (2024). Metabolic targeting of cancer associated fibroblasts overcomes T-cell exclusion and chemoresistance in soft-tissue sarcomas. Nat Commun.

[44] Domingues HS (2016). Oligodendrocyte, astrocyte, and microglia crosstalk in myelin development, damage, and repair. Front Cell Dev Biol.

[45] Herenius MMJ (2011). Monocyte migration to the synovium in rheumatoid arthritis patients treated with adalimumab. Ann Rheum Dis.

[46] Thurlings RM (2009). Monocyte scintigraphy in rheumatoid arthritis: the dynamics of monocyte migration in immune-mediated inflammatory disease. PLoS One.

[47] Hasegawa T (2024). Macrophages and nociceptor neurons form a sentinel unit around fenestrated capillaries to defend the synovium from circulating immune challenge. Nat Immunol.

[48] Kurowska-Stolarska M, Alivernini S (2017). Synovial tissue macrophages: friend or foe?. RMD Open.

[49] Fang C (2024). TREM2 promotes macrophage polarization from M1 to M2 and suppresses osteoarthritis through the NF-κB/CXCL3 axis. Int J Biol Sci.

[50] Orecchioni M (2019). Macrophage polarization: different gene signatures in M1(LPS+) vs. classically and M2(LPS-) vs. alternatively activated macrophages. Front Immunol.

[51] Strizova Z (2023). M1/M2 macrophages and their overlaps - myth or reality?. Clin Sci (Lond).

[52] Buechler MB (2021). Fibroblast-macrophage reciprocal interactions in health, fibrosis, and cancer. Immunity.

[53] Culemann S (2019). Locally renewing resident synovial macrophages provide a protective barrier for the joint. Nature.

[54] Jaitin DA (2019). Lipid-associated macrophages control metabolic homeostasis in a Trem2-dependent manner. Cell.

[55] Cang Z (2023). Screening cell-cell communication in spatial transcriptomics via collective optimal transport. Nat Methods.

[56] Heesters BA (2014). Follicular dendritic cells: dynamic antigen libraries. Nat Rev Immunol.

[57] Bery AI (2022). Role of tertiary lymphoid organs in the regulation of immune responses in the periphery. Cell Mol Life Sci.

[58] Gregorio A (2007). Lymphoid neogenesis in juvenile idiopathic arthritis correlates with ANA positivity and plasma cells infiltration. Rheumatology (Oxford).

[59] Sato Y (2023). The roles of tertiary lymphoid structures in chronic diseases. Nat Rev Nephrol.

[60] Baccin C (2019). Combined single-cell and spatial transcriptomics reveal the molecular, cellular and spatial bone marrow niche organization. Nat Cell Biol.

[61] Croft AP (2019). Distinct fibroblast subsets drive inflammation and damage in arthritis. Nature.

[62] Del Rey MJ (2009). Human inflammatory synovial fibroblasts induce enhanced myeloid cell recruitment and angiogenesis through a hypoxia-inducible transcription factor 1alpha/vascular endothelial growth factor-mediated pathway in immunodeficient mice. Arthritis Rheum.

[63] Nerviani A (2020). A pauci-immune synovial pathotype predicts inadequate response to TNFα-blockade in rheumatoid arthritis patients. Front Immunol.

[64] Mizoguchi F (2018). Functionally distinct disease-associated fibroblast subsets in rheumatoid arthritis. Nat Commun.

[65] Müller-Ladner U (1996). Synovial fibroblasts of patients with rheumatoid arthritis attach to and invade normal human cartilage when engrafted into SCID mice. Am J Pathol.

[66] Muhl L (2020). Single-cell analysis uncovers fibroblast heterogeneity and criteria for fibroblast and mural cell identification and discrimination. Nat Commun.

[67] Ando K (2019). Peri-arterial specification of vascular mural cells from naïve mesenchyme requires Notch signaling. Development.

[68] Nakano M (2022). Distinct transcriptome architectures underlying lupus establishment and exacerbation. Cell.

[69] Qin S (1998). The chemokine receptors CXCR3 and CCR5 mark subsets of T cells associated with certain inflammatory reactions. J Clin Invest.

[70] Martini G (2005). CXCR3/CXCL10 expression in the synovium of children with juvenile idiopathic arthritis. Arthritis Res Ther.

[71] Lim HY (2018). Hyaluronan receptor LYVE-1-expressing macrophages maintain arterial tone through hyaluronan-mediated regulation of smooth muscle cell collagen. Immunity.

[73] Bombardieri M (2017). Ectopic lymphoid neogenesis in rheumatic autoimmune diseases. Nat Rev Rheumatol.

[74] Danaher P (2024). Childhood-onset lupus nephritis is characterized by complex interactions between kidney stroma and infiltrating immune cells. Sci Transl Med.

[75] Cohen S (2024). A phase 1, randomized, double-blind, placebo-controlled, single- and multiple-dose escalation study to evaluate the safety and pharmacokinetics/pharmacodynamics of PF-06835375, a C-X-C chemokine receptor type 5 directed antibody, in patients with systemic lupus erythematosus or rheumatoid arthritis. Arthritis Res Ther.

[76] Lan F (2025). GZMK-expressing CD8^+^ T cells promote recurrent airway inflammatory diseases. Nature.

[77] Donado CA (2025). Granzyme K activates the entire complement cascade. Nature.

[78] Jin S (2024). CellChat for systematic analysis of cell-cell communication from single-cell transcriptomics. Nat Protoc.

[79] Efremova M (2020). CellPhoneDB: inferring cell-cell communication from combined expression of multi-subunit ligand-receptor complexes. Nat Protoc.

[80] Vento-Tormo R (2018). Single-cell reconstruction of the early maternal-fetal interface in humans. Nature.

[81] Noël F (2021). Dissection of intercellular communication using the transcriptome-based framework ICELLNET. Nat Commun.

