# Supplementary material for: Spatial transcriptomics reveals immune-stromal crosstalk within the synovium of patients with juvenile idiopathic arthritis
Source: JCI Insight. 2025 Nov 21;11(1):e198074. doi: 10.1172/jci.insight.198074 (PMC12890527; doi:10.1172/jci.insight.198074)
Supplement: Supplemental data [file jciinsight-11-198074-s125.pdf]

Collection of sample and processing

Synovial tissue samples were obtained via ultrasound-guided biopsy using a Quick-Core needle. Six to eight tissue fragments were fixed in formalin for subsequent paraffin embedding and histological processing.

FFPE tissue sections were dried in an oven, deparaffinized through a xylene gradient, and de-crosslinked according to the manufacturer's instructions (10x Genomics protocol CG000578 and CG000580). The Xenium Prime RNA probe set (10x Genomics) was hybridized overnight, ligated, and amplified using rolling circle amplification (10x Genomics protocol CG000760). All incubations are performed using reagents provided by 10x Genomics on a VeritiPro Thermocycler (Applied Biosystems). Autofluorescence was quenched and morphology stains were added, including nuclear, cytoplasmic, and membrane stains for improved cell segmentation (10x Genomics). Slides were then loaded onto the Xenium instrument for whole slide tissue overview scanning and region selection. Regions containing tissue were selected and the onboard cyclic decoding and imaging was initiated over a 72-120-hour period. Data was processed on the instrument after image acquisition, performing probe deconvolution, cell segmentation.

Histology Assessment

FFPE tissue sections were used for histological analysis. Hematoxylin and eosin (H&E)-stained synovial biopsy sections were examined, and a pathologist assigned Krenn inflammatory infiltrate score.

Xenium data preprocessing

Raw Xenium datasets were loaded and processed individually using the Seurat v5 R package (1). Data was read using a customized function, capturing gene expression matrices and spatial coordinates, including centroids and segmentations. After loading, each dataset was filtered to retain cells with detected genes between 200 and 5000 and total transcripts less than 25,000.

SCTransform was applied individually to each sample for normalization and variance stabilization. Subsequently, the nine samples were merged into a single Seurat object, followed by another round of SCTransform (2) to ensure integrated normalization. We then performed principal component analysis (PCA) with 20 principal components (PCs) on the merged dataset with 2000 variable features and performed Harmony (3) integration (lambda=1, theta=2, and sigma=0.1) to correct for batch effects by different slides and samples. Uniform Manifold Approximation and Projection (UMAP) were applied post-integration. Cell cycle phase was inferred by cell types using CellCycleScoring function in the Seurat R package.

Cell type identification

We constructed a shared nearest neighbor (SNN) graph based on the first 20 harmonized PCs and identified initial broad cell types based on clustering results with the Louvain algorithm at resolution 0.20. Broad clusters were categorized into (1) stromal tissue cells (fibroblasts and endothelial cells), (2) myeloid cells, (3) T cells/ILCs (including NK cells), and (4) B/plasma cells based on the expression of canonical markers. Cells identified as belonging to these broad cell types were then extracted into separate datasets for further analysis. Each cell type underwent a second round of SCTransform normalization, PCA, Harmony integration, and UMAP dimensionality reduction. Louvain clustering was again performed separately on these subsets, with resolutions tailored specifically for each cell type (T/ILCs: 1.00, B/plasma: 0.40, myeloid: 0.20, stromal tissue: 0.60) to achieve refined subpopulation identification. The effect of batch correction was assessed using Local Inverse Simpson's Index (LISI) (3). Cell clusters were annotated based on the expression of canonical marker genes. To identify top10 marker genes characteristic of each cluster, we applied the wilcoxauc function from the presto R package (4) to normalized expression data. Differential expression was first assessed between each cluster and all remaining cells using the Wilcoxon rank-sum test. In addition, to account for potential confounding from broad lineage differences, we repeated the differential expression analysis

within each cell type, comparing each subcluster to other subclusters. Following differential expression analysis, we selected the top 10 genes per cluster ranked by log fold change and reported in **Supplementary Table 2**.

Identification of cell populations that are associated with specific clinical variables

To identify cell states with disease-specific spatial organization, we implemented a CNA (5). Briefly, CNA quantifies the relationship between each cell based on transcriptome similarity, allowing detection of transcriptome neighborhoods covary with sample-level conditions such as disease status (e.g., CRP level). We first defined a SNN graph using harmonized PCs. This matrix was then used to calculate a neighborhood correlation score.

Integration of spatial transcriptomics and GWAS data using gsMap

To identify genetically relevant spatially localized cell types within the synovium, we applied gsMap, a computational framework that integrates spatial transcriptomic profiles with GWAS summary statistics (6). In the gsMap framework, each cell was embedded into a low-dimensional representation that captured both molecular and spatial context. Gene specificity scores were computed for each cell based on the learned latent space, representing how specifically each gene is expressed in a given cell's spatial context. Then, these gene specificity scores were integrated with GWAS signals for JIA (GWAS catalog: GCST90010715) (7). Using precomputed LD scores and SNP weights from the 1000 Genomes Project European reference panel (Phase 3), gsMap estimated the heritability enrichment for each spatial cell by modeling the association between GWAS summary statistics and spatially defined LD scores.

Identifying spatial niches

To identify spatial niches, we took two complementary approaches. First, we utilized a method inspired by Goltsev et al. (8) and He et al. (9), implemented in Seurat's BuildNicheAssay function.

This method defines the `local neighborhood` for each cell by considering its `k.neighbor` spatially closest neighbors and counts occurrences of each cell type present within this neighborhood. Cells sharing similar neighborhood compositions are grouped into spatial niches using k-means clustering. We made slight modification of this function to identify corresponding niches across different slides and used k.neighbor=30. To identify biologically interpretable niche clusters within identified niches, we then constructed a cell-type composition matrix across all identified niches. This matrix was derived by calculating the proportion of each annotated cell type in each spatial niche, excluding “mixed”, “proliferating”, and “muscle cell” clusters to reduce noise. PCA was performed on the normalized composition matrix to reduce dimensionality and capture the major sources of variation in niche composition. We determined the optimal number of PCs to retain based on the cumulative proportion of variance explained, selecting the smallest number of PCs that accounted for at least 30% of the total variance, resulting in the top 3 PCs in total for the downstream process. To classify niches into broader biologically meaningful categories, we applied k-means clustering to the PCA-reduced data. The optimal number of clusters was selected based on the average silhouette width, which evaluates the compactness and separation of clusters. The k=4 that maximized the average silhouette score was chosen as the optimal value. Based on the dominant cell types in each cluster and biological interpretability, we manually annotated each niche cluster as one of the following: Stromal niche, Myeloid + Stromal cell niche, Myeloid+T cell niche, or T + B/plasma cell niche. To assess pathway activity within spatially defined niches, we applied the escape R package (v2.2.3) (10) to perform single-cell gene set enrichment analysis (11). We used a curated collection of gene sets from the Molecular Signatures Database (MSigDB) (12), including Hallmark gene sets (13) and KEGG pathway gene sets (14). Gene sets were filtered to retain only those genes expressed in the dataset. Enrichment scores were computed on the normalized expression matrix using escape.matrix using “ssGSEA” method (11) with 5000 random background groups and a minimum gene set size of 5. Scores

were subsequently normalized using the performNormalization function to account for differences in gene detection rate, ensuring comparability across cells.

Secondly, we developed a custom method that performs spatial neighborhood enrichment analyses. For each slide, spatial coordinates (`centroids`) of cells were obtained, and the nearest neighbors for each cell were identified using the FindNeighbors function in Seurat with a parameter `neighbors.k = 30`. We then constructed an adjacency matrix representing spatial relationships among cells by combining nearest neighbor information across slides using block diagonal matrices. The observed spatial enrichment of cell type interactions was quantified using a custom function, which calculates occurrences of each pairwise cell type interaction within spatial neighborhoods. To assess the statistical significance of these observed interactions, 500 permutation tests were performed by randomly shuffling cluster labels across cells. Z-scores were calculated to measure how significantly the observed neighborhood compositions deviated from randomized expectations.

Module score calculation for pro-inflammatory macrophage, LYVE-1^{+} perivascular macrophage, and CX3CR1^{+} lining macrophage signature

To quantify the pro-inflammatory polarization state in macrophages, we computed a module score based on a curated set of pro-inflammatory marker genes. The gene set was derived from published studies that define transcriptional programs associated with pro-inflammatory signature (15, 16), including *ZBP1*, *RSAD2*, *TAP2*, *VCAM1*, *BATF*, *CD86*, *SERPINE1*, *PVR*, *CXCL16*, *CCND2*, *PDPN*, *CCRL2*, *FLNB*, *MAP3K5*, *IL17RA*, *RBPMS*, *INHBA*, *TRAF1*, *NFKB2*, *VASP*, *PTGES*, *IFNAR2*, *IL1RN*, *CD274*, *NOTCH1*, *IFI35*, *IFIT2*, *ICAM1*, *IL12B*, *IL12A*, *CD14*, *ITGA5*, *EIF2AK2*, *PILRA*, *CFLAR*, *TNIP1*, *TNFRSF1B*, *TLR2*, *MMP14*, *OAS3*, *ADORA2A*, *JDP2*, *IRF7*, *MITD1*, *CD40*, *CXCL9*, *GRAMD1A*, *EBI3*, *SOCS3*, *PSTPIP2*, *ACP5*, *CD38*, *IL15RA*, *SERPINB2*, *STAT1*, *ARG2*, *SYK*, *GCH1*, *STAT2*, *HCK*, *MET*, *HDC*, *SNX10*, *ITGAL*, *PTGS2*, *IFIH1*, *TRIM25*,

and *JAK2*. For each cell, we calculated the average expression level of the pro-inflammatory marker genes using the row-wise mean across the normalized expression matrix.

In the same way, we computed a module score for the LYVE-1^{+} signature using genes upregulated in FOLR^{high}LYVE-1^{+} macrophages, listed in Supplementary Data 1 of the original publication (17). Genes not detected in our dataset were excluded. The final FOLR^{high}LYVE-1^{+} macrophage gene set used for scoring included the following genes detected in our dataset: *F13A1, SLC40A1, STAB1, PLTP, FOLR2, LGMN, HMOX1, COLEC12, GAS6, MRC1, DAB2, MAF, THBD, EMP1, LILRB5, CTSC, PMP22, PEPD, SLCO2B1, GPR34, CSF1R, TSPAN4, CD14, NINJ1, CD4, MEF2C, RBPJ, MAFB, IFI16, CLTC, ABL2, CD59, CTSL, EIF4E, FCGR2B, ATF3, CD99, UCP2, LAMP1,* and *FCGR2A.*

To investigate the presence of barrier-forming macrophage phenotypes in our dataset, we curated a gene signature associated with CX3CR1^{+} lining macrophages, as reported by Culemann et al.(18). The signature was derived from the set of marker genes enriched in CX3CR1^{+} macrophages (upregulated genes in lining vs. interstitial macrophage in Fig. 3B and marker genes of cluster 3 in Fig. 3C-D of the original paper), and corresponding mouse gene symbols were converted to human orthologs using Ensembl annotation and manual curation. After intersecting with our measured genes, a module score was calculated using the following genes: *FAT3, CX3CR1, ADCYAP1R1, CDH23, ALDOA, FABP7, HTR2B, SIRPB1, TSPAN18, NPNT, OLFML3, and VSIG4.*

Simulation analysis for method evaluation

To evaluate the performance of our spatial neighborhood enrichment analyses, we employed simulation data generated using custom R scripts. Two spatial configurations, termed “concentric circle” and “layer”, were simulated to assess the sensitivity and robustness of our method in detecting predefined spatial neighborhood patterns. For each simulation scenario, we generated datasets comprising 1,500 cells randomly distributed across a defined spatial domain (800 × 800

μm). Cells were assigned into 10 distinct cell types. To create spatially enriched interactions, we specifically arranged two cell types to exhibit spatial proximity while randomly distributing the remaining cell types within the spatial field.

In the concentric circle scenario, cells belonging to the anchor cell type (reference) were primarily concentrated within a central circular area, whereas neighboring cells were positioned to form an outer ring surrounding this central region at a fixed distance. Additional random noise was introduced to mimic realistic spatial variation. Cells from other cell types were uniformly randomly distributed across the entire spatial domain.

In the layer scenario, spatial enrichment was structured along layer patterns. Reference cells were positioned along a straight horizontal line, with neighboring cells located in parallel at a fixed vertical offset. As with the circle scenario, random spatial noise was added to replicate biological heterogeneity.

Using these synthetic spatial distributions, we performed neighborhood enrichment analyses to validate the sensitivity of our method to detect known spatial relationships. We then assessed spatial enrichment of cell type interactions by computing z-scores from 500 permutations by randomly shuffling cluster labels across cells. Significant cell-cell enrichments were determined based on Benjamini-Hochberg FDR-corrected p-values.

Colocalization score

To investigate spatial proximity at the single-cell/spot level, we computed a colocalization score that measures the local co-occurrence of two specified cell clusters. This analysis complements the spatial neighborhood approach by providing insights at the resolution of individual cells rather than at the broader cell-type level. For each cell, we first identified neighboring cells within a defined radius using spatial coordinates derived from imaging data. Specifically, we utilized the nn2 function in the RANN R package (19) to find neighbors around a cell of the anchor cell type within a radius of 10 μm. We then assessed whether both anchor and target clusters were present

among these neighboring cells. A binary score was assigned to each cell, indicating the simultaneous presence (score of 1) or absence (score of 0) of both clusters within its immediate spatial neighborhood. To account for broader spatial context and to smooth local colocalization signals across the tissue, we implemented a random walk, inspired by covarying neighborhood analysis (5), based on the adjacency matrix derived from the spatial neighbor network. The adjacency matrix was constructed using spatial coordinates from multiple samples, integrated using a block-diagonal matrix. In the random walk step, the local colocalization scores were propagated iteratively through neighboring cells, weighted by their spatial connectivity, until convergence was determined by monitoring changes in the kurtosis of the score distribution. This iterative diffusion process was limited to a maximum of 15 steps to ensure computational efficiency while achieving stable estimates of spatial co-occurrence patterns. The final result provided a quantitative measure for each spatial spot, reflecting its degree of spatial co-occurrence with the specified clusters in its immediate neighborhood, allowing precise identification of spatially defined microenvironments and potential cell-cell interactions.

Public spatial transcriptomic dataset acquisition and preprocessing

Public spatial transcriptomic datasets from 10X Genomics Xenium platform were utilized for benchmarking purposes. The human breast dataset and mouse brain dataset were downloaded from the 10X Genomics website and preprocessed using the Seurat framework.

For each dataset, spatial gene expression matrices and spatial coordinate metadata were imported. Cells with zero total gene counts were excluded from downstream analysis. Gene expression values were normalized using SCTransform and PCA was performed. SNN graphs were constructed, and clustering was performed using the Louvain algorithm with resolution at 0.6. For the human breast dataset, cell types were annotated based on cluster-specific marker gene expression derived using FindAllMarkers function, followed by manual inspection and comparison with known markers for each tissue. The mouse brain dataset was annotated,

following instruction in the Seurat tutorial, using the RCTD (Robust Cell Type Decomposition) pipeline (20) with a reference atlas of Allen Brain subclass annotations downloaded from https://www.dropbox.com/s/cuowvm4vrf65pvq/allen_cortex.rds?dl=1. The spatial object was processed with create.RCTD() and run.RCTD() in “doublet” mode, and predicted cell types were assigned to each spot based on highest posterior probability.

We applied a spatial neighborhood analysis approach to evaluate non-random co-occurrence of cell types. We computed a Z-score-based enrichment matrix from observed cell-type neighbor frequencies compared to a null distribution of 500 spatial permutations. Neighborhoods were defined by k-nearest neighbors (k = 30). To assess spatial colocalization score between specific cell types at the single-spot level, we used a search radius of 30 μm and up to 15 neighborhood expansion steps.

Ligand–receptor analysis

To systematically identify ligand–receptor interactions at single-spot resolution, we performed ligand–receptor analyses by samples using COMMOT (21), a Python-based toolkit designed for spatial communication inference. COMMOT integrates spatial coordinates and transcriptomic data to infer ligand–receptor signaling interactions between spots. An integrated ligand–receptor database was constructed by combining human ligand–receptor pairs from CellChat (22) and CellPhoneDB v4.0 databases (22, 23). Spatial ligand–receptor communication was computed using the spatial_communication function with the merged database. We set the spatial distance threshold to 200 μm and enabled analysis of heteromeric ligand–receptor complexes. COMMOT analysis generated spatial communication scores indicating the strength of ligand–receptor interactions from sender and receiver perspectives for each spatial spot.

Flow cytometry and intracellular cytokine staining

Synovial fluid mononuclear cells were isolated from patients with oligo/poly JIA using Ficoll-Paque density gradient centrifugation. Cells were resuspended in complete RPMI medium and stimulated for 6 hours at 37°C with 50 ng/mL PMA and 1 µg/mL ionomycin (Biolegend), 10 ng/mL IL-12 (PEPRO-TECH, catalogue number: 200-12), 50 ng/mL IL-15 (PEPRO-TECH, catalogue number: 200-15) and 50 ng/mL IL-18 (MBL, catalogue number: B001-5) with last 4 hours in the presence of 10 µg/mL brefeldin A (BioLegend, catalogue number: 420601). After stimulation, cells were washed, stained with Zombie NIR Fixable viability dye (Biolegend, catalogue number: 423106), and surface-stained with fluorochrome-conjugated antibodies against CD3 (Biolegend, clone OKT3,), CD4 (Biolegend, clone SK3), CD8 (Biolegend, clone SK1), CD45 (Biolegend, clone 2D1), CD56 (Biolegendclone 5.1.H11), and TCRγδ (Biolegend, clone 3C10). Cells were then fixed and permeabilized using the Cytofix/Cytoperm kit (BD Biosciences, catalogue number: 554714) and stained intracellularly with anti–IFN-γ antibody (Biolegend, clone 4S.B3). Flow cytometry was performed on a Cytek Aurora and data were analyzed using FlowJo v10. All antibodies were diluted 1 to 100.

Identification of tertiary lymphoid structures

To systematically identify TLS, we developed a custom pipeline leveraging the colocalization score described above. First, we calculated the colocalization scores specifically between broad B-cell and T-cell populations. Next, we used spatial coordinates (centroids) obtained from each slide to construct spatial adjacency graphs for individual cells with positive colocalization scores for B-cell and T-cell populations, enabling a more efficient search for relevant interactions compared to scanning the entire spatial transcriptomic dataset. For this step, nearest neighbor cells were identified using a radius-based nearest neighbor search, the nn2 function in the RANN R package, with parameters set to a radius of 50 µm and the maximum number of nearest neighbors set to 200. Subsequently, an undirected spatial adjacency graph was constructed where each node represents a cell, and edges connect spatially proximate cells identified in the

radius-based search. Using the igraph package, connected components within this graph were identified, each representing clusters of spatially associated cells. Clusters meeting predefined thresholds—specifically, those containing at least 20 B cells and T cells—were annotated as candidate TLS. The resulting candidate TLS identified through this computational pipeline were subsequently validated by a single pathologist who was blinded to computational analysis results.

To investigate the spatial organization of immune cells relative to B cells within TLS, we calculated the Euclidean distance to the nearest B cell for each cell within a TLS region. These distances were binned in 5 μm intervals from 0 to 300 μm. Within each bin, we computed the proportion of cells for each annotated immune cluster relative to the total number of cells in that cluster. We further estimated the spatial decay rate with distance. For each cluster, we examined how the proportion of cells decreased as a function of distance from the nearest TLS B cell. This was done by fitting a simple linear regression model with distance as the predictor and the cluster-specific proportion as the outcome. The slope of this regression line was interpreted as the decay rate, expressed as the average percentage change per micrometer. Negative values indicate that the cluster is enriched close to TLS B cells and declines with increasing distance, while values close to zero reflect a more uniform distribution. Ninety-five percent confidence intervals were obtained from the regression fit to quantify the uncertainty of each estimate.

Immunohistochemistry staining

An antigen retrieval was performed using a Decloaking Chamber (Biocare Medical) at 95 degrees for 1 hour in Tris/EDTA antigen retrieval buffer (Agilent). They were then stained consecutively with primary antibodies for MERTK (Abcam, clone ab52968), TREM2 (Invitrogenclone TREM2/7210), CD68 (DAKO, clone KP1), CD3E (Leica, clone LN10), CD8 (DAKO, clone C8/144B), and IL1B (Cell Signaling Technologies, clone 3A6) using cyclic multispectral fluorescence immunohistochemistry. Briefly, the slides were blocked, incubated with primary antibodies, followed by horseradish peroxidase (HRP)-conjugated secondary antibody polymer,

and HRP-reactive OPAL fluorescent reagents using a Bond RX autostainer (Leica). The slides were stripped between each stain with heat treatment in an antigen retrieval buffer. Spectral references and unstained control images were measured and inForm software v3.0 was used to create a multispectral library reference. Whole slide scans were collected using the 20x objective with a 0.5 μm resolution and were spectrally unmixed with PhenoImager HT v2.0.0 software.

Comparison with RA CITE-seq data

Cellular Indexing of Transcriptomes and Epitopes by sequencing (CITE-seq) data from RA synovial tissue were retrieved from previously published dataset (24). The dataset was integrated with the Xenium-derived JIA data using the StabMap method (25). Integration was based on variable mRNA features in the JIA Xenium dataset and present in the RA CITE-seq dataset. UMAP embedding was performed on the integrated PCA. Cell-type labels were transferred from the RA CITE-seq data to the JIA Xenium data through k-nearest neighbors (k-NN) classification, training the model on the RA dataset with known subtype labels. This integration process was carried out independently by each broad cell type. As for cell clusters defined by integrated PCA, only clusters with sufficient cell numbers (n ≥ 300 cells) were considered for downstream interpretation.

Supplementary references

1. Hao Y, et al. Dictionary learning for integrative, multimodal and scalable single-cell analysis. *Nat Biotechnol*. 2024;42(2):293–304.

2. Hafemeister C, Satija R. Normalization and variance stabilization of single-cell RNA-seq data using regularized negative binomial regression. *Genome Biol*. 2019;20(1):296.

3. Korsunsky I, et al. Fast, sensitive and accurate integration of single-cell data with Harmony. *Nature Methods*. 2019;16(12):1289–1296.

4. GitHub - immunogenomics/presto: Fast Wilcoxon and auROC [Internet]. *GitHub*. https://github.com/immunogenomics/presto. Accessed April 8, 2025.

5. Reshef YA, et al. Co-varying neighborhood analysis identifies cell populations associated with phenotypes of interest from single-cell transcriptomics. *Nature Biotechnology*. 2021;40(3):355–363.

6. Song L, et al. Spatially resolved mapping of cells associated with human complex traits. *Nature*. 2025;1–10.

7. López-Isac E, et al. Combined genetic analysis of juvenile idiopathic arthritis clinical subtypes identifies novel risk loci, target genes and key regulatory mechanisms. *Ann Rheum Dis*. 2021;80(3):321–328.

8. Goltsev Y, et al. Deep Profiling of Mouse Splenic Architecture with CODEX Multiplexed Imaging. *Cell*. 2018;174(4):968–981.e15.

9. He S, et al. High-plex imaging of RNA and proteins at subcellular resolution in fixed tissue by spatial molecular imaging. *Nat Biotechnol*. 2022;40(12):1794–1806.

10. Borcherding N, et al. Mapping the immune environment in clear cell renal carcinoma by single-cell genomics. *Commun Biol*. 2021;4(1):122.

11. Barbie DA, et al. Systematic RNA interference reveals that oncogenic KRAS-driven cancers

require TBK1. *Nature*. 2009;462(7269):108–112.

12. Subramanian A, et al. Gene set enrichment analysis: a knowledge-based approach for interpreting genome-wide expression profiles. *Proc Natl Acad Sci U S A*. 2005;102(43):15545–15550.

13. Liberzon A, et al. The Molecular Signatures Database (MSigDB) hallmark gene set collection. *Cell Syst*. 2015;1(6):417–425.

14. Kanehisa M, et al. KEGG for taxonomy-based analysis of pathways and genomes. *Nucleic Acids Res*. 2023;51(D1):D587–D592.

15. Orecchioni M, et al. Macrophage Polarization: Different Gene Signatures in M1(LPS+) vs. Classically and M2(LPS-) vs. Alternatively Activated Macrophages. *Front Immunol*. 2019;10:1084.

16. Strizova Z, et al. M1/M2 macrophages and their overlaps - myth or reality? *Clin Sci (Lond)*. 2023;137(15):1067–1093.

17. Alivernini S, et al. Distinct synovial tissue macrophage subsets regulate inflammation and remission in rheumatoid arthritis. *Nature Medicine*. 2020;26(8):1295–1306.

18. Culemann S, et al. Locally renewing resident synovial macrophages provide a protective barrier for the joint. *Nature*. 2019;572(7771):670–675.

19. GitHub - jefferislab/RANN: R package providing fast nearest neighbour search (wraps ANN library) [Internet]. *GitHub*. https://github.com/jefferislab/RANN. Accessed March 20, 2025.

20. Cable DM, et al. Robust decomposition of cell type mixtures in spatial transcriptomics. *Nature Biotechnology*. 2021;40(4):517–526.

21. Cang Z, et al. Screening cell–cell communication in spatial transcriptomics via collective optimal transport. *Nature Methods*. 2023;20(2):218–228.

22. Jin S, Plikus MV, Nie Q. CellChat for systematic analysis of cell–cell communication from single-cell transcriptomics. *Nature Protocols*. 2024;20(1):180–219.

23. Efremova M, et al. CellPhoneDB: inferring cell–cell communication from combined expression of multi-subunit ligand–receptor complexes. *Nature Protocols*. 2020;15(4):1484–1506.

24. Zhang F, et al. Deconstruction of rheumatoid arthritis synovium defines inflammatory subtypes. *Nature*. 2023;623(7987):616–624.

25. Ghazanfar S, Guibentif C, Marioni JC. Stabilized mosaic single-cell data integration using unshared features. *Nat Biotechnol*. 2024;42(2):284–292.

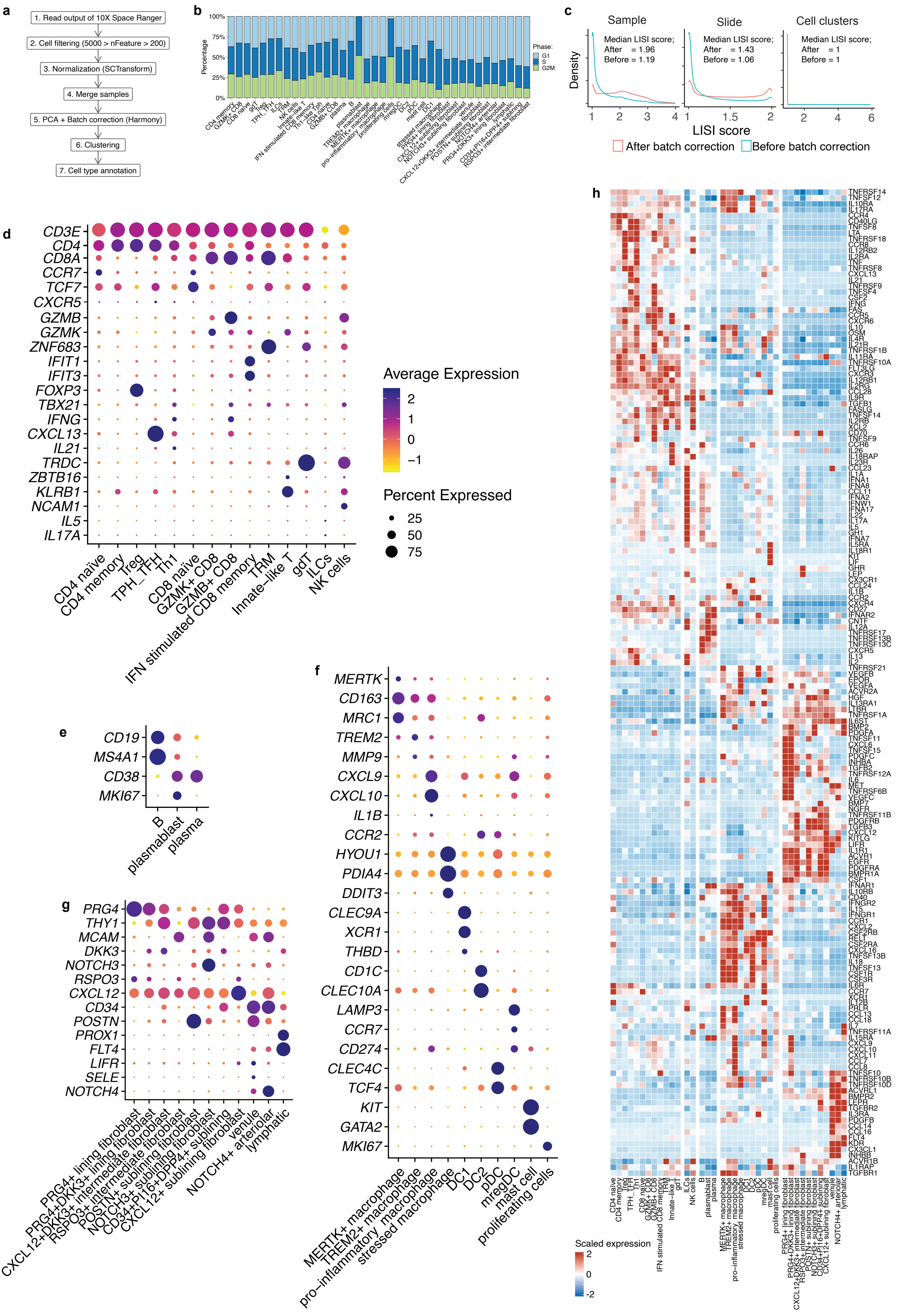
a
1. Read output of 10X Space Ranger
2. Cell filtering (5000 > nFeature > 200)
3. Normalization (SCTransform)
4. Merge samples
5. PCA + Batch correction (Harmony)
6. Clustering
7. Cell type annotation
b
Percentage
100%
75%
50%
25%
0%
Phase:
G1
S
G2M
CD4 memory
GZMK+ CD8
CD8 naive
gdT
Treg
TPH_TFH
ILCs
TRM
NK cells
Innate-like T
IFN stimulated CD8 memory
Th1
CD4 naive
GZMB+ CD8
plasma
B
plasmablast
TREM2+ macrophage
MERTK+ macrophage
pro-inflammatory macrophage
proliferating cells
mregDC
DC2
pDC
DC1
mast cell
stressed macrophage
PRG4+ lining fibroblast
CXCL12+DKK3+ intermediate fibroblast
NOTCH3+ sublining fibroblast
POSTN+ sublining fibroblast
venule
CXCL12+ sublining fibroblast
NOTCH4+ arteriolar
PRG4+DKK3+ lining fibroblast
lymphatic
CD34+P16+DPP4+ sublining
RSPO3+ intermediate fibroblast
c
Density
Sample
Median LISI score;
After = 1.96
Before = 1.19
Slide
Median LISI score;
After = 1.43
Before = 1.06
Cell clusters
Median LISI score;
After = 1
Before = 1
LISI score
After batch correction
Before batch correction
d
CD3E
CD4
CD8A
CCR7
TCF7
CXCR5
GZMB
GZMK
ZNF683
IFIT1
IFIT3
FOXP3
TBX21
IFNG
CXCL13
IL21
TRDC
ZBTB16
KLRB1
NCAM1
IL5
IL17A
CD4 naive
CD4 memory
Treg
TPH_TFH
Th1
CD8 naive
GZMK+ CD8
GZMB+ CD8
IFN stimulated CD8 memory
TRM
Innate-like T
gdT
ILCs
NK cells
Average Expression
2
1
0
−1
Percent Expressed
25
50
75
e
CD19
MS4A1
CD38
MKI67
B
plasmablast
plasma
f
MERTK
CD163
MRC1
TREM2
MMP9
CXCL9
CXCL10
IL1B
CCR2
HYOU1
PDIA4
DDIT3
CLEC9A
XCR1
THBD
CD1C
CLEC10A
LAMP3
CCR7
CD274
CLEC4C
TCF4
KIT
GATA2
MKI67
MERTK+ macrophage
TREM2+ macrophage
pro−inflammatory macrophage
stressed macrophage
DC1
DC2
pDC
mregDC
mast cell
proliferating cells
g
PRG4
THY1
MCAM
DKK3
NOTCH3
RSPO3
CXCL12
CD34
POSTN
PROX1
FLT4
LIFR
SELE
NOTCH4
PRG4+ lining fibroblast
PRG4+DKK3+ lining fibroblast
CXCL12+DKK3+ intermediate fibroblast
RSPO3+ intermediate fibroblast
POSTN+ sublining fibroblast
NOTCH3+ sublining fibroblast
CD34+P16+DPP4+ sublining
CXCL12+ sublining fibroblast
NOTCH4+ venule
arteriolar
lymphatic
h
TNFRSF14
TNFSF12
IL10RA
IL17RA
CCR4
CD40LG
TNFSF8
LTA
TNFRSF18
CCR8
IL12RB2
IL2RA
TNF
TNFRSF8
CXCL13
IL21
TNFRSF9
TNFSF4
CSF2
IFNG
FAS
CCR5
CXCR6
IL10
OSM
IL4R
IL21R
TNFRSF1B
IL11RA
TNFRSF10A
FLT3LG
CXCR3
IL12RB1
IL2RG
CCL28
IL9R
TGFB1
FASLG
TNFSF14
IL2RB
XCL2
CD70
TNFSF9
CCR6
IL26
IL18RAP
IL23R
CCL23
IL1A
IFNA1
IFNA8
CCL11
IFNA2
IFNW1
IFNA17
IL22
IL17A
IL5
GH1
IFNA7
IL5RA
IL18R1
KIT
LIF
GHR
LEP
CX3CR1
CCL24
IL1B
CCR2
CXCR4
CD27
IFNAR2
CNTF
IL12A
TNFRSF17
TNFRSF13B
TNFRSF13C
CXCR5
IL13
IL2
TNFRSF21
VEGFB
EPOR
VEGFA
ACVR2A
HGF
IL13RA1
LTBR
TNFRSF1A
IL6ST
BMP2
PDGFA
TNFSF11
CXCL6
TNFSF15
PDGFC
INHBA
TGFB2
TNFRSF12A
IL6
MET
TNFRSF6B
VEGFC
BMP7
NGFR
TNFRSF11B
PDGFRB
TGFB3
CXCL12
KITLG
LIFR
IL1R1
ACVR1
EGFR
PDGFRA
BMPR1A
CSF1
IFNAR1
IL10RB
CD40
IFNGR2
IL15
IFNGR1
CCR1
CXCL2
CSF2RB
RELT
CSF2RA
CXCL16
TNFSF13B
IL18
TNFSF13
CSF1R
CSF3R
IL6R
CCR7
XCR1
IL12B
PRLR
CCL13
CCL18
IL7
TNFRSF11A
IL15RA
CXCL9
CXCL10
CXCL11
CCL7
CCL8
TNFSF10
TNFRSF10B
TNFRSF10D
ACVRL1
BMPR2
LEPR
TGFBR2
IL3RA
PDGFB
CCL14
CCL16
FLT4
KDR
CX3CL1
INHBB
ACVR1B
IL1RAP
TGFBR1
Scaled expression
2
0
-2

Supplemental Figure 1: Preprocessing steps for spatial transcriptome data. a, Workflow of preprocessing steps. **b**, Inferred cell cycle phase. **c**, Local Inverse Simpson's Index (LISI) scores to measure mixture levels on samples, slides, and cell clusters. After batch effect correction, the mixture level of samples and slides are significantly reduced compared to before correction (Wilcoxon test $p < 0.01$). **d-g,** Dot plot showing marker gene expressions in identified cell clusters, for T/ILC cluster (**d**), B/plasma cluster (**e**), Myeloid cluster (**f**), and stromal cluster (**g**). **h**, Heatmap showing Z-scored pseudo-bulk expression profiles of cytokine and cytokine receptor genes across 38 synovial cell states. Genes were selected from the MSigDB C2 curated collection (M9809). Only genes detected in more than 3% of cells within any cluster were retained (n = 168). Expression values were aggregated at the cluster level and row-scaled to highlight relative expression patterns across cell populations.

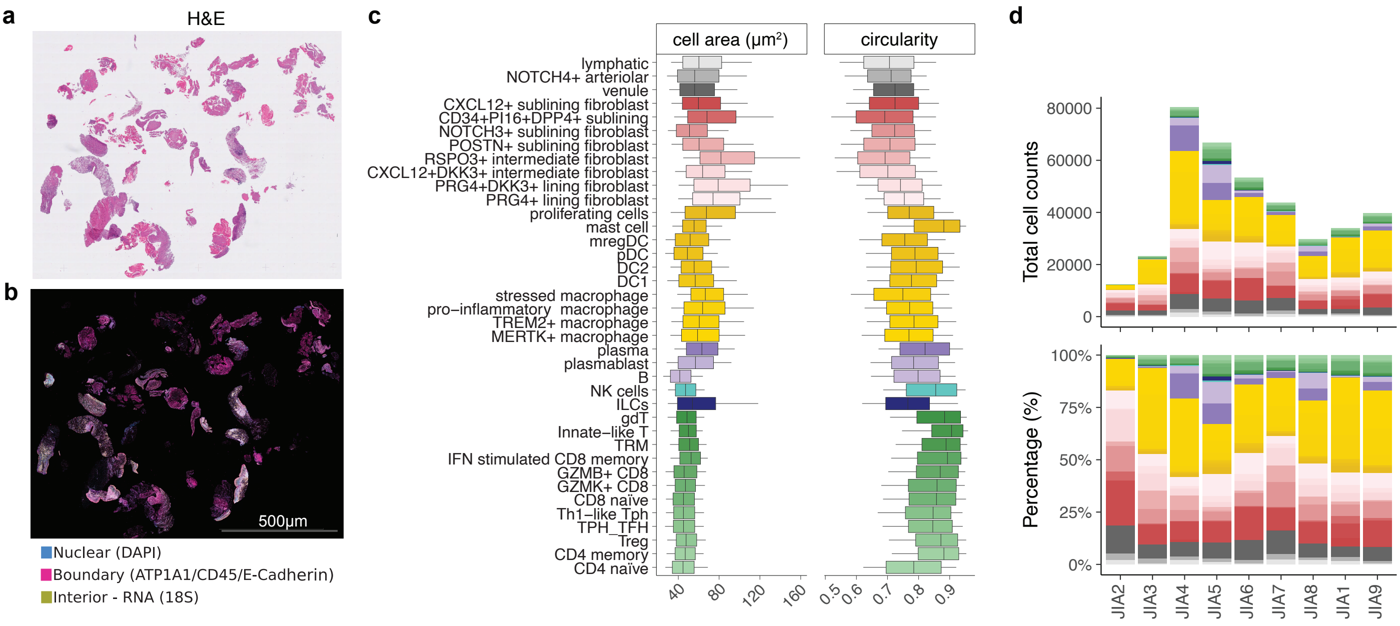

a
H&E
b
500μm
Nuclear (DAPI)
Boundary (ATP1A1/CD45/E-Cadherin)
Interior - RNA (18S)
c
cell area (μm²)
circularity
lymphatic
NOTCH4+ arteriolar
venule
CXCL12+ sublining fibroblast
CD34+PI16+DPP4+ sublining
NOTCH3+ sublining fibroblast
POSTN+ sublining fibroblast
RSPO3+ intermediate fibroblast
CXCL12+DKK3+ intermediate fibroblast
PRG4+DKK3+ lining fibroblast
PRG4+ lining fibroblast
proliferating cells
mast cell
mregDC
pDC
DC2
DC1
stressed macrophage
pro−inflammatory macrophage
TREM2+ macrophage
MERTK+ macrophage
plasma
plasmablast
B
NK cells
ILCs
gdT
Innate−like T
TRM
IFN stimulated CD8 memory
GZMB+ CD8
GZMK+ CD8
CD8 naïve
Th1−like Tph
TPH_TFH
Treg
CD4 memory
CD4 naïve
40
80
120
160
0.5
0.6
0.7
0.8
0.9
d
Total cell counts
80000
60000
40000
20000
0
Percentage (%)
100%
75%
50%
25%
0%
JIA2
JIA3
JIA4
JIA5
JIA6
JIA7
JIA8
JIA1
JIA9

Supplemental Figure 2: Representative histological and spatial transcriptomic visualization of JIA synovium. a-b, Hematoxylin and eosin (H&E) staining of a representative synovial tissue sample (JIA4) (**a**), and corresponding spatial transcriptomic image visualized using Xenium Explorer (**b**). In panel **b**, Nuclear signals (DAPI) are shown in blue, cell boundary markers (ATP1A1, CD45, and E-cadherin) in magenta, and RNA signal (18S rRNA) in yellow. **c,** Box plots show the distribution of cell area (left) and circularity (right) across annotated cell populations. Circularity was calculated as $(4 \times \pi \times \text{Area}) / (\text{Perimeter}^2)$, with values closer to 1 indicating rounder cells. Box plots showing the median, interquartile range, and 1.5× interquartile range (IQR) whiskers. **d,** Composition of cell-types across samples.

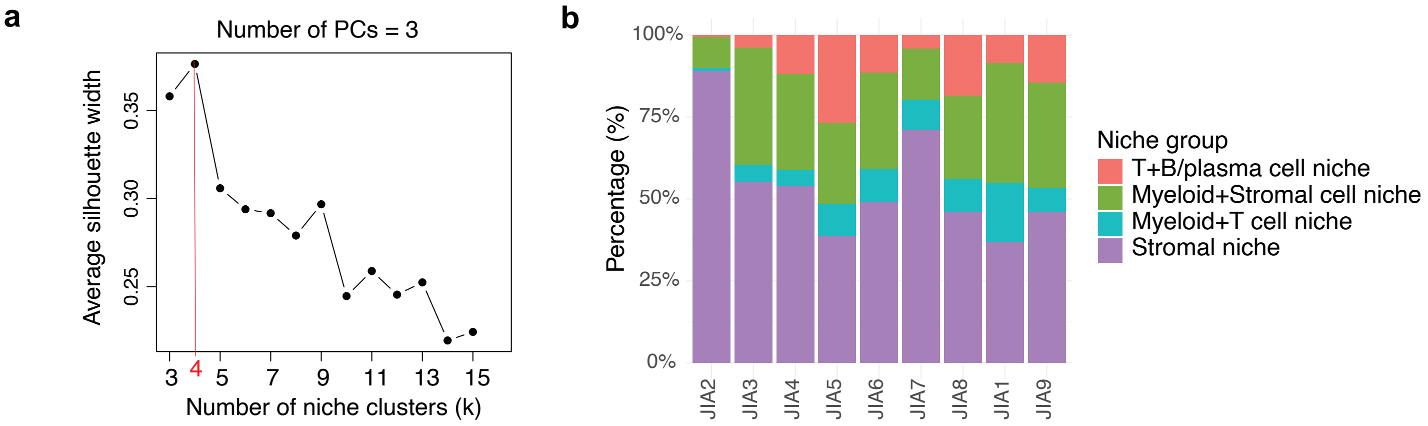
a
Number of PCs = 3
Average silhouette width
0.25
0.30
0.35
3
4
5
7
9
11
13
15
Number of niche clusters (k)
b
Percentage (%)
100%
75%
50%
25%
0%
JIA2
JIA3
JIA4
JIA5
JIA6
JIA7
JIA8
JIA1
JIA9
Niche group
T+B/plasma cell niche
Myeloid+Stromal cell niche
Myeloid+T cell niche
Stromal niche

Supplemental Figure 3: Identification and distribution of niche clusters across JIA synovial tissues. a, Silhouette analysis for determining the optimal number of niche clusters. Based on PCA of the niche × cell type composition matrix, the silhouette width was calculated across a range of cluster numbers ($k = 3$ to 15). The optimal number of clusters was determined to be $k = 4$ (red line), based on the maximum average silhouette width, using the first 3 principal components (explaining ≥30% variance). **b,** Proportion of each annotated niche group across individual JIA samples. Each bar represents the percentage composition of niche groups within a single patient sample.

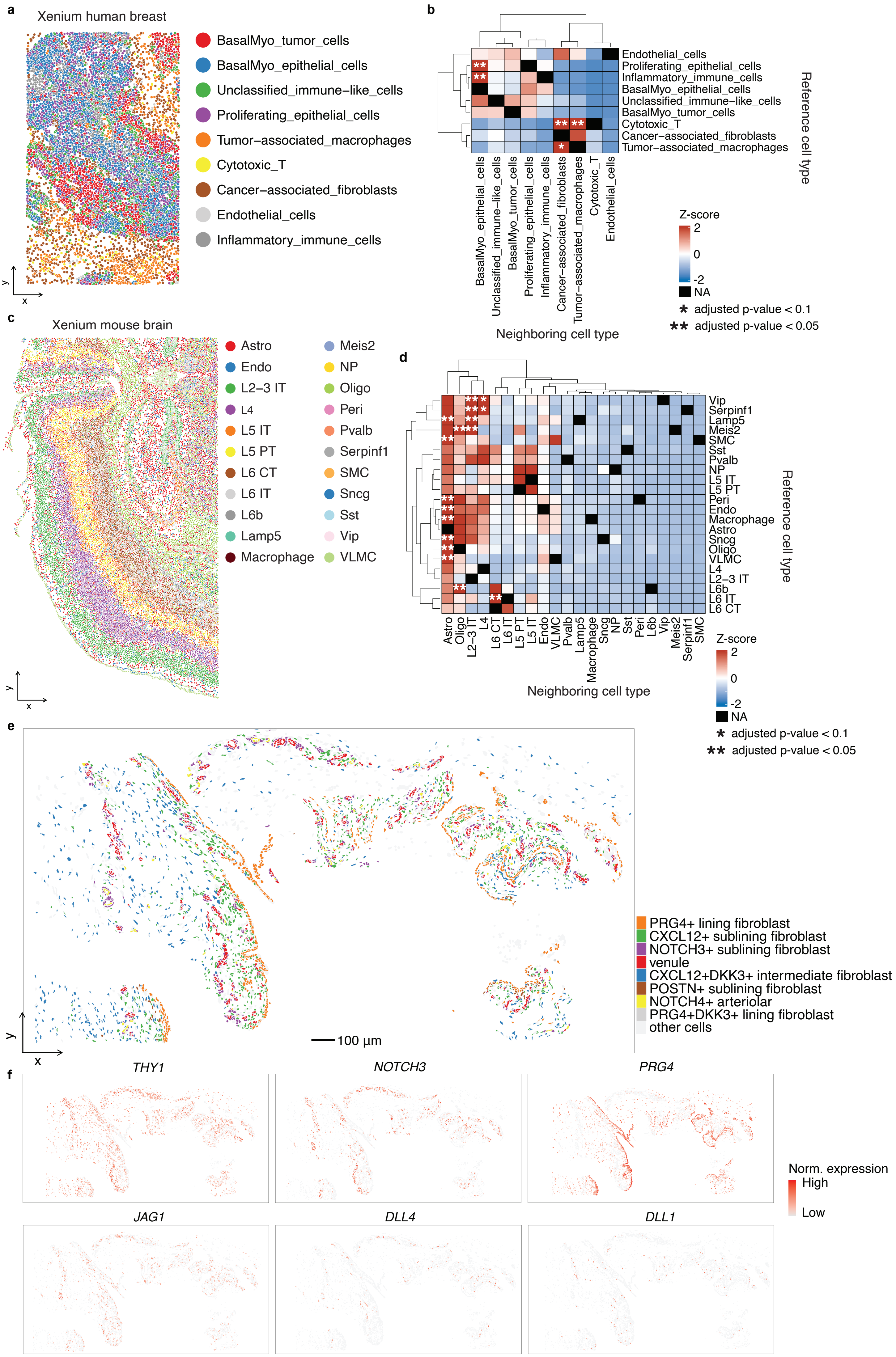
a
Xenium human breast
BasalMyo_tumor_cells
BasalMyo_epithelial_cells
Unclassified_immune–like_cells
Proliferating_epithelial_cells
Tumor–associated_macrophages
Cytotoxic_T
Cancer–associated_fibroblasts
Endothelial_cells
Inflammatory_immune_cells
b
Reference cell type
Neighboring cell type
Z-score
NA
* adjusted p-value < 0.1
** adjusted p-value < 0.05
c
Xenium mouse brain
Astro
Endo
L2–3 IT
L4
L5 IT
L5 PT
L6 CT
L6 IT
L6b
Lamp5
Macrophage
Meis2
NP
Oligo
Peri
Pvalb
Serpinf1
SMC
Sncg
Sst
Vip
VLMC
d
Reference cell type
Neighboring cell type
Z-score
NA
* adjusted p-value < 0.1
** adjusted p-value < 0.05
e
PRG4+ lining fibroblast
CXCL12+ sublining fibroblast
NOTCH3+ sublining fibroblast
venule
CXCL12+DKK3+ intermediate fibroblast
POSTN+ sublining fibroblast
NOTCH4+ arteriolar
PRG4+DKK3+ lining fibroblast
other cells
100 µm
f
THY1
NOTCH3
PRG4
JAG1
DLL4
DLL1
Norm. expression
High
Low

Supplemental Figure 4: Benchmarking the pipeline of spatial neighborhood enrichment analysis using public spatial transcriptome datasets. **a**, Spatial distribution of annotated cell types within the human breast cancer tissue dataset derived from the Xenium spatial transcriptome technology. Each color corresponds to a distinct cell type. **b**, Heatmap of spatial neighborhood enrichment in the human breast cancer tissue. The color gradient represents z-scores indicating significant spatial interactions between pairs of cell types, with red denoting enriched (positively associated) spatial proximity and blue representing depleted (negatively associated) interactions. Statistical significance is annotated as * (adjusted p-value < 0.1 by Benjamini-Hochberg method) and ** (adjusted p-value < 0.05). **c**, Spatial distribution of annotated cell types within the mouse brain tissue dataset derived from the Xenium spatial transcriptome technology. Each color corresponds to a distinct cell type. **d**, Heatmap of spatial neighborhood enrichment in the mouse brain tissue. **e**, Representative spatial coordinates of a identified niche of sub-lining fibroblasts and endothelial cells, matching pathological images. Cells are colored according to their cluster assignments. **f**. Spatial expression patterns of fibroblast-related and Notch signaling-related genes within the identified niche location.

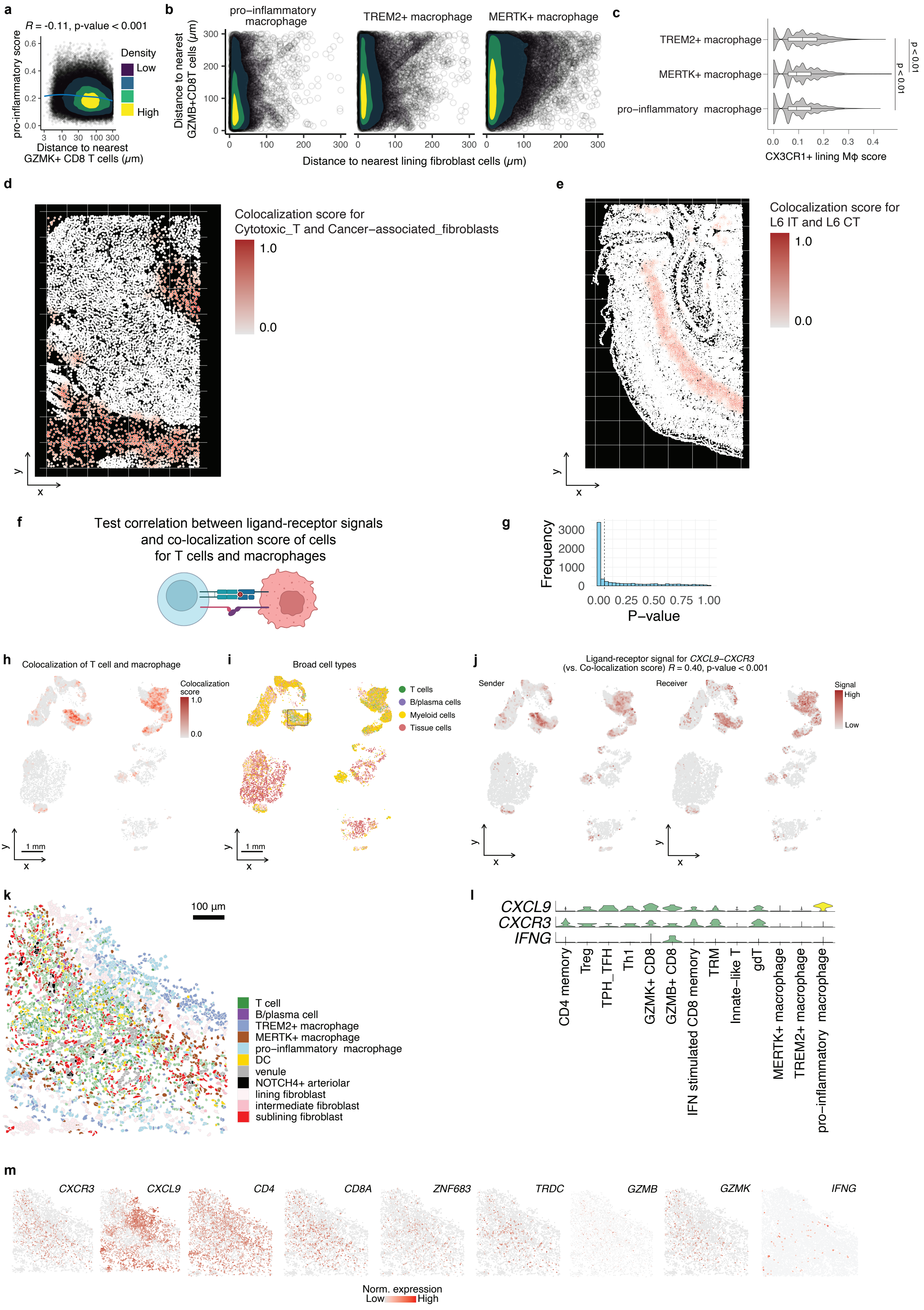

a
R = -0.11, p-value < 0.001
pro-inflammatory score
Density
Low
High
Distance to nearest GZMK+ CD8 T cells (μm)
b
pro−inflammatory macrophage
TREM2+ macrophage
MERTK+ macrophage
Distance to nearest GZMB+CD8T cells (μm)
Distance to nearest lining fibroblast cells (μm)
c
TREM2+ macrophage
MERTK+ macrophage
pro−inflammatory macrophage
p < 0.01
p < 0.01
CX3CR1+ lining Mϕ score
d
Colocalization score for Cytotoxic_T and Cancer−associated_fibroblasts
1.0
0.0
e
Colocalization score for L6 IT and L6 CT
1.0
0.0
f
Test correlation between ligand-receptor signals and co-localization score of cells for T cells and macrophages
g
Frequency
P−value
h
Colocalization of T cell and macrophage
Colocalization score
1 mm
i
Broad cell types
T cells
B/plasma cells
Myeloid cells
Tissue cells
1 mm
j
Ligand-receptor signal for CXCL9–CXCR3
(vs. Co-localization score) R = 0.40, p-value < 0.001
Sender
Receiver
Signal
High
Low
k
100 µm
T cell
B/plasma cell
TREM2+ macrophage
MERTK+ macrophage
pro−inflammatory macrophage
DC
venule
NOTCH4+ arteriolar
lining fibroblast
intermediate fibroblast
sublining fibroblast
l
CXCL9
CXCR3
IFNG
CD4 memory
Treg
TPH_TFH
Th1
GZMK+ CD8
GZMB+ CD8
IFN stimulated CD8 memory
TRM
Innate−like T
gdT
MERTK+ macrophage
TREM2+ macrophage
pro−inflammatory macrophage
m
CXCR3
CXCL9
CD4
CD8A
ZNF683
TRDC
GZMB
GZMK
IFNG
Norm. expression
Low
High

Supplemental Figure 5: Benchmarking the pipeline of colocalization scoring analysis using public spatial transcriptome datasets. A, Correlation between pro-inflammatory module scores of individual macrophage cells (each point) (y-axis) and distance to the *GZMK*+ CD8 T cells (x-axis). Color-filled contours represent two-dimensional kernel density estimates. Spearman's correlation coefficient and p-values are shown. **b,** Density plots depict the distances of individual macrophage cells (each point) to the nearest lining fibroblast cells (x-axis) and *GZMB*+ CD8 T cells (y-axis) by macrophage subtypes. Color-filled contours represent two-dimensional kernel density estimates. **c**, Violin plots showing the distribution of CX3CR1+ lining macrophage module scores across macrophage subtypes. **d-e**, Colocalization scores distribution between Cytotoxic T cells and Cancer-associated fibroblasts in the human breast cancer (**d**) and L6 IT and L6CT in the mouse brain (**e**) spatial transcriptome datasets which were introduced in Supplemental Figure 4. **f**, Shema of the analytical approach used to correlate ligand–receptor interactions with spatial colocalization scores for T cells and macrophages. **g**, Distribution of p-values from Spearman's correlation tests between ligand–receptor signaling strength and colocalization scores between macrophages and T cells. **h**, Spatial heatmap of colocalization scores between T cells and macrophages. **i**, Spatial map of broad cell type annotation including T cells, myeloid cells, B/plasma cells, and tissue cells. **j**, Spatial distribution of predicted CXCL9–CXCR3 ligand-receptor. **k**, Zoom-in of a representative region showing identified niche of T cells and macrophages. Cells are colored according to their cluster assignments. **l**, Violin plots showing the expression levels of T- and macrophage-related genes across different cell clusters. **m**, Spatial visualization of the expression levels of T- and macrophage-related genes at the matched histopathological locations. Color scale indicates normalized expression levels.

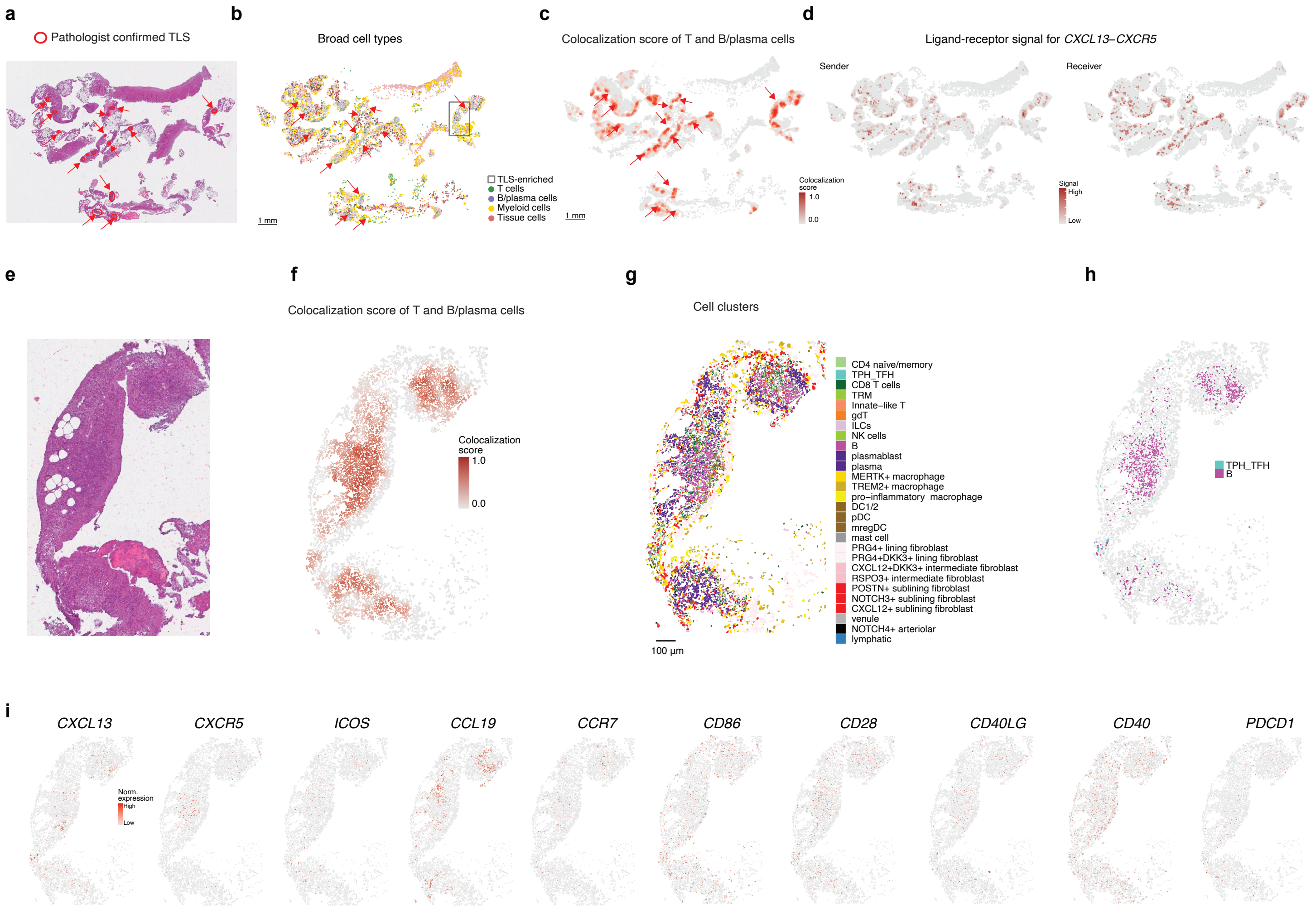

a
Pathologist confirmed TLS
b
Broad cell types
TLS-enriched
T cells
B/plasma cells
Myeloid cells
Tissue cells
1 mm
c
Colocalization score of T and B/plasma cells
Colocalization score
1.0
0.0
1 mm
d
Ligand-receptor signal for CXCL13–CXCR5
Sender
Receiver
Signal
High
Low
e
f
Colocalization score of T and B/plasma cells
Colocalization score
1.0
0.0
g
Cell clusters
CD4 naïve/memory
TPH_TFH
CD8 T cells
TRM
Innate–like T
gdT
ILCs
NK cells
B
plasmablast
plasma
MERTK+ macrophage
TREM2+ macrophage
pro–inflammatory macrophage
DC1/2
pDC
mregDC
mast cell
PRG4+ lining fibroblast
PRG4+DKK3+ lining fibroblast
CXCL12+DKK3+ intermediate fibroblast
RSPO3+ intermediate fibroblast
POSTN+ sublining fibroblast
NOTCH3+ sublining fibroblast
CXCL12+ sublining fibroblast
venule
NOTCH4+ arteriolar
lymphatic
100 µm
h
TPH_TFH
B
i
CXCL13
CXCR5
ICOS
CCL19
CCR7
CD86
CD28
CD40LG
CD40
PDCD1
Norm. expression
High
Low

Supplemental Figure 6: Identified TLS-clusters in JIA synovium. a, H&E staining images of synovial tissue, illustrating representative TLS as identified by a blinded pathologist. **b**, Spatial distribution of T and B cells. Scale bar, 1 mm. The highlighted black square indicates the TLS-enriched region. **c**, Colocalization scores for T and B cells. **d**, Spatial *CXCL13*-*CXCR5* interaction signals. **e**, H&E staining image of zoomed-in of the area with the highest TLS density highlighted in panel **b. f-h**, Same region as in panel **e**, colored by T and B cell colocalization score (**f**) and fine-scale cell cluster assignments (**g-h**). **i**, Spatial expression patterns of TLS-associated genes.

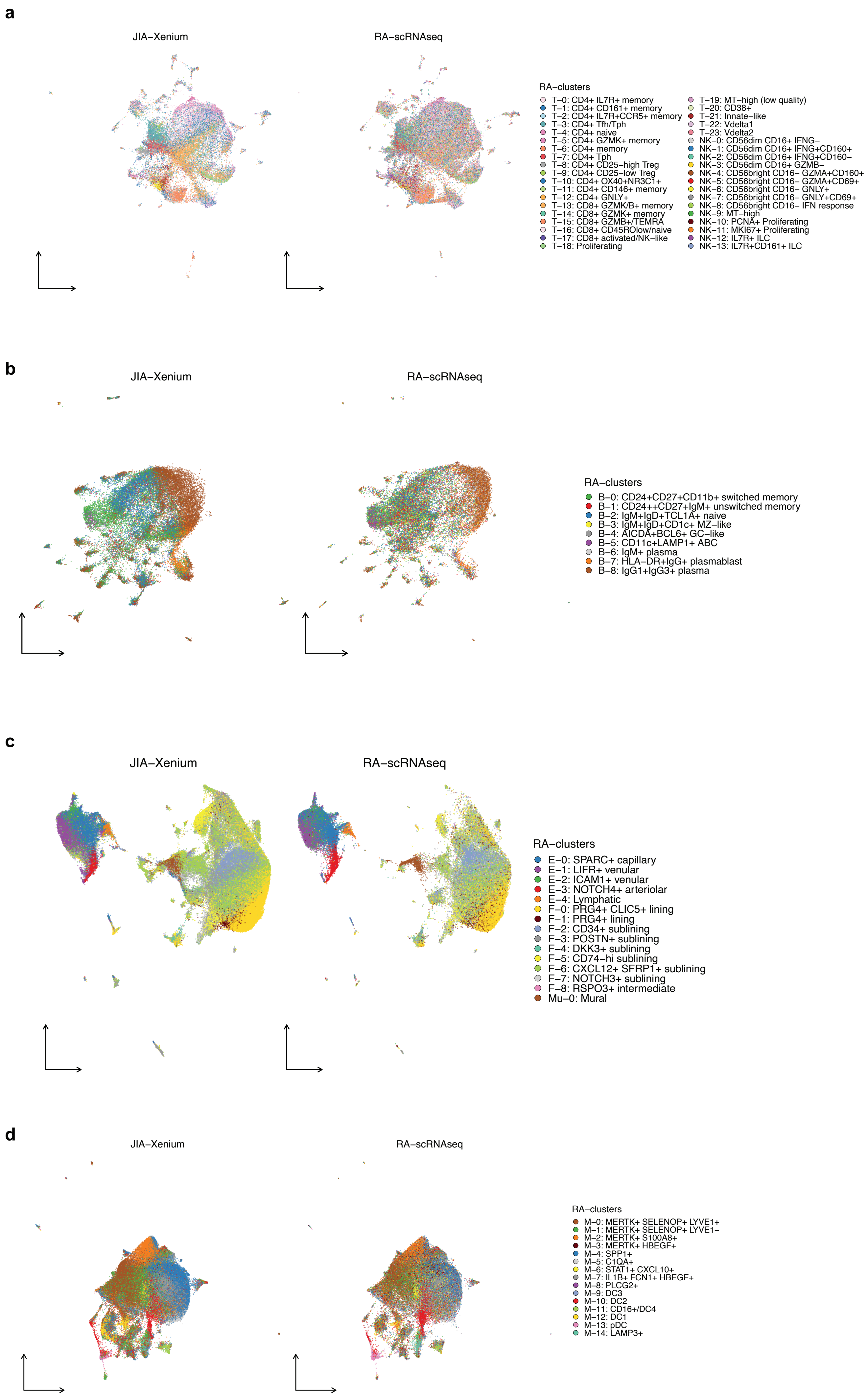
a
JIA–Xenium
RA–scRNAseq
RA–clusters
T–0: CD4+ IL7R+ memory
T–1: CD4+ CD161+ memory
T–2: CD4+ IL7R+CCR5+ memory
T–3: CD4+ Tfh/Tph
T–4: CD4+ naive
T–5: CD4+ GZMK+ memory
T–6: CD4+ memory
T–7: CD4+ Tph
T–8: CD4+ CD25–high Treg
T–9: CD4+ CD25–low Treg
T–10: CD4+ OX40+NR3C1+
T–11: CD4+ CD146+ memory
T–12: CD4+ GNLY+
T–13: CD8+ GZMK/B+ memory
T–14: CD8+ GZMK+ memory
T–15: CD8+ GZMB+/TEMRA
T–16: CD8+ CD45ROlow/naive
T–17: CD8+ activated/NK–like
T–18: Proliferating
T–19: MT–high (low quality)
T–20: CD38+
T–21: Innate–like
T–22: Vdelta1
T–23: Vdelta2
NK–0: CD56dim CD16+ IFNG–
NK–1: CD56dim CD16+ IFNG+CD160+
NK–2: CD56dim CD16+ IFNG+CD160–
NK–3: CD56dim CD16+ GZMB–
NK–4: CD56bright CD16– GZMA+CD160+
NK–5: CD56bright CD16– GZMA+CD69+
NK–6: CD56bright CD16– GNLY+
NK–7: CD56bright CD16– GNLY+CD69+
NK–8: CD56bright CD16– IFN response
NK–9: MT–high
NK–10: PCNA+ Proliferating
NK–11: MKI67+ Proliferating
NK–12: IL7R+ ILC
NK–13: IL7R+CD161+ ILC
b
JIA–Xenium
RA–scRNAseq
RA–clusters
B–0: CD24+CD27+CD11b+ switched memory
B–1: CD24++CD27+IgM+ unswitched memory
B–2: IgM+IgD+TCL1A+ naive
B–3: IgM+IgD+CD1c+ MZ–like
B–4: AICDA+BCL6+ GC–like
B–5: CD11c+LAMP1+ ABC
B–6: IgM+ plasma
B–7: HLA–DR+IgG+ plasmablast
B–8: IgG1+IgG3+ plasma
c
JIA–Xenium
RA–scRNAseq
RA–clusters
E–0: SPARC+ capillary
E–1: LIFR+ venular
E–2: ICAM1+ venular
E–3: NOTCH4+ arteriolar
E–4: Lymphatic
F–0: PRG4+ CLIC5+ lining
F–1: PRG4+ lining
F–2: CD34+ sublining
F–3: POSTN+ sublining
F–4: DKK3+ sublining
F–5: CD74–hi sublining
F–6: CXCL12+ SFRP1+ sublining
F–7: NOTCH3+ sublining
F–8: RSPO3+ intermediate
Mu–0: Mural
d
JIA–Xenium
RA–scRNAseq
RA–clusters
M–0: MERTK+ SELENOP+ LYVE1+
M–1: MERTK+ SELENOP+ LYVE1–
M–2: MERTK+ S100A8+
M–3: MERTK+ HBEGF+
M–4: SPP1+
M–5: C1QA+
M–6: STAT1+ CXCL10+
M–7: IL1B+ FCN1+ HBEGF+
M–8: PLCG2+
M–9: DC3
M–10: DC2
M–11: CD16+/DC4
M–12: DC1
M–13: pDC
M–14: LAMP3+

Supplemental Figure 7: Cell type mapping between JIA Xenium spatial transcriptomics and RA synovial scRNA-seq data across immune and stromal compartments. a-d: UMAP projections of JIA synovium (Xenium, left) and RA synovium (scRNA-seq, right) showing StabMap-based label transfer for major immune and stromal compartments. Each cell is colored according to the RA scRNA-seq-derived reference cluster annotation transferred to Xenium cells using k-nearest neighbor (kNN) mapping in a shared latent space. **a**: T/ILCs, **b**: B cell and plasma cell subsets, **c**: Fibroblast and endothelial cell subsets, and **d**: Myeloid cell subsets.

Supplementary Table 1: Clinical features of the patients

Patient	JIA 1	JIA 2	JIA 3	JIA 4	JIA 5	JIA 6	JIA 7	JIA 8	JIA 9
**Sex**	F	F	M	F	M	F	F	F	F
**Age (years)**	7	10	10	2	8	7	18	9	8
**Ethnicity (Hispanic)**	No	No	Yes	Yes	No	No	No	Yes	No
**Disease Duration (years)**	0.5	6	3	0.2	0.5	4	16	0.2	0.5
**Time from flare onset to biopsy (years)**	0.1	0.1	0.3	0.2	0.5	0.2	0.1	0.2	0.5
**JIA type**	Oligo	Oligo	Poly	Oligo	Poly	Poly	Oligo	Oligo	Oligo
**Source of Synovium**	Knee	Knee	Knee	Knee	Knee	Knee	Knee	Knee	Knee
**Prior treatment**	Steroid injection	Steroid injection	MTX, LEF, ADA	None	None	Steroid injection, MTX	MTX, ETA, ADA	None	MTX
**Systemic treatment at the time of biopsy**	None	MTX	LEF, ADA	None	None	MTX	none	None	MTX
**Uveitis**	Negative	Negative	Positive	Negative	Negative	Negative	Positive	Negative	Negative
**ANA**	Positive	Positive	Positive	Positive	Positive	Positive	Negative	Positive	Positive
**RF**	Negative	Negative	Negative	Negative	Negative	Negative	Negative	Negative	Negative
**CRP (mg/L)**	0	35	29	11	0	0	0	0	0
**Krenn score (total)**	6	2	5	8	7	6	8	4	6
**Krenn lining**	2	0	1	2	2	2	3	1	2
**Krenn inflammation**	2	1	2	3	3	2	3	1	2

**Krenn stroma**	2	1	2	3	2	2	2	2	2

Oligo: oligoarticular juvenile idiopathic arthritis, Poly: polyarticular juvenile idiopathic arthritis, MTX: methotrexate, LEF: leflunomide, ETA: etanercept, ADA: adalimumab, ANA: antinuclear antibody, RF: rheumatoid factor, CRP: C-reactive protein
